# Twelve-Year Analysis of NO_2_ Concentration Measurements at Belisario Station (Quito, Ecuador) Using Statistical Inference Techniques

**DOI:** 10.3390/s20205831

**Published:** 2020-10-15

**Authors:** Wilmar Hernandez, Alfredo Mendez

**Affiliations:** 1 Facultad de Ingeniería y Ciencias Aplicadas, Universidad de Las Américas, Quito 170125, Ecuador; 2 Departamento de Matemática Aplicada a las Tecnologías de la Información y las Comunicaciones, ETS de Ingeniería y Sistemas de Telecomunicación, Universidad Politécnica de Madrid, 28031 Madrid, Spain; alfredo.mendez@upm.es

**Keywords:** statistical inference, classic analysis, nonparametric analysis, robust analysis, classic confidence interval, nonparametric confidence interval, bootstrap confidence interval, robust confidence interval, classification and categorization of NO_2_ concentration measurements

## Abstract

In this paper, a robust analysis of nitrogen dioxide (NO_2_) concentration measurements taken at Belisario station (Quito, Ecuador) was performed. The data used for the analysis constitute a set of measurements taken from 1 January 2008 to 31 December 2019. Furthermore, the analysis was carried out in a robust way, defining variables that represent years, months, days and hours, and classifying these variables based on estimates of the central tendency and dispersion of the data. The estimators used here were classic, nonparametric, based on a bootstrap method, and robust. Additionally, confidence intervals based on these estimators were built, and these intervals were used to categorize the variables under study. The results of this research showed that the NO_2_ concentration at Belisario station is not harmful to humans. Moreover, it was shown that this concentration tends to be stable across the years, changes slightly during the days of the week, and varies greatly when analyzed by months and hours of the day. Here, the precision provided by both nonparametric and robust statistical methods served to comprehensively proof the aforementioned. Finally, it can be concluded that the city of Quito is progressing on the right path in terms of improving air quality, because it has been shown that there is a decreasing tendency in the NO_2_ concentration across the years. In addition, according to the Quito Air Quality Index, most of the observations are in either the desirable level or acceptable level of air pollution, and the number of observations that are in the desirable level of air pollution increases across the years.

## 1. Introduction

Nitrogen dioxide (NO_2_) is a yellowish brown toxic and irritant gas, and together with nitric oxide (NO) is known as nitrogen oxides (NOx) [1,2]. Several negative health effects are attributed to continued exposure to this pollutant, such as: acute bronchitis, asthma, reduced lung capacity, allergies, eye and mucous membrane irritation [3]. It is a secondary precursor to ozone (O_3_) and particulate matter PM_2.5_ [4,5].

NO_2_ is mainly formed from the oxidation of NO as a result of combustion in vehicle engines and combustion plants with oxygen (O_2_) from the air [1]:

(1) $$2\mathrm{NO}\;+{\;O}_{2}\;↔\;2{\mathrm{NO}}_{2}$$

To a lesser extent atmospheric NO_2_ comes from natural sources such as volcanic eruptions, atmospheric electric shocks, and biomass combustion. It is a very strong oxidant; it reacts with water (H_2_O) and OH radical producing nitric acid (HNO_3_) [6]:

(2) $$3{\mathrm{NO}}_{2}\;+{\;H}_{2}O\;\to \;2{\mathrm{HNO}}_{3}\;+\;\mathrm{NO}$$

(3) $$\mathrm{OH}\;+{\;\mathrm{NO}}_{2}\;\to {\;\mathrm{HNO}}_{3}$$

The particles that form from this acid can be suspended in the air or fall like acid rain [1]. The World Health Organization (WHO) recommends a daily NO_2_ concentration of $0.11\;\mathrm{ppm}\;\left(200\;\mu g/m^{3}\right)$ average of 1 h once a year and $0.023\;\mathrm{ppm}\;\left(40\;\mu g/m^{3}\right)$ as an annual arithmetic average to preserve health [7]. NO_2_ production volumes correspond to vehicular traffic; being this way, they are generally higher in cities than in the countryside.

The negative effects that air pollution due to NO_2_ concentrations has on human health and the way in which this pollution is produced, lead to the need for an in-depth statistical analysis of the behavior of this pollutant in environments where its generation is more likely. In this sense, the city of Quito, the capital of Ecuador, is a good place to carry out this analysis taking into account the information collected by measurement instruments for more than a decade.

In this paper, Quito was chosen to carry out the analysis mentioned above because this city is an example of how the environmental impact of vehicular traffic, traffic jams and poor fuel quality can affect pollution levels. Likewise, the growth of the city, of industrial zones and the fact that Quito is surrounded by a mountain range are factors that also influence the concentration of pollutants in this city [8].

Taking into account what has been said above, the main objective of this paper is to carry out the robust analysis of NO_2_ concentration measurements in one the most important air-quality monitoring stations in Quito. To this end, the Belisario air-quality monitoring station [9] was chosen to carry out the research, and 12 years of NO_2_ concentration measurements (i.e., from 1 January 2008 to 31 December 2019) were analyzed. Other pollutants that are also measured at Belisario station are the following: CO (carbon monoxide), SO_2_ (sulfur dioxide), O_3_ (ozone), and PM_2.5_ (fine particulate matter = particles with a diameter less than or equal to 2.5 μm) [8,10]. However, it is important to mention that this paper focused on the robust analysis of the behavior of NO_2_ concentration at Belisario station, because NO_2_ is a pollutant that is predominant in the appearance and duration of health problems [3]. In fact, the study of toxicity mechanisms due to NO_2_ in humans is a subject of great interest to the international scientific community [3]. For example, it is of great interest to understand the relationship between exposure to NO_2_ and sensitivity to viral infections, among other things.

The analysis performed in this paper was aimed at estimating the central tendency and dispersion of the data, grouping and classifying data, and determining similarities and differences between data. Furthermore, the analysis was performed by using both robust and nonrobust confidence intervals, and classical, nonparametric, resampling and robust analysis methodologies were used [11,12,13,14,15].

A research in which the statistical analysis of NO_2_ concentration measurements was carried out is shown in [16]. In [16], in order to perform air-quality monitoring measurements in an area of Puerto Rico, low-cost particulate matter (PM_2.5_) and NO_2_ sensors were placed across eight locations of that area. In [16], the NO_2_ concentration measurements were taken from October 2016 to February 2017, spatial and temporal trends of PM_2.5_ and NO_2_ were analyzed, and the measurements were collected using the U.S. Environmental Protection Agency (EPA)-designed Citizen Science Air Monitor (CSAM).

The analysis carried out in [16] was based on the use of classical inference methods, where linear regressions were used to normalize each low-cost sensor and weather station with a reference signal. Additionally, the correlation function was used to find the degree of linear dependence between sensor measurements and the median of the reference signal. Moreover, in [16] the Pearson coefficient and the coefficient of divergence were used to explore the spatial variability between CSAM locations. Furthermore, the coefficient of variance was used to calculate the precision between sensors.

In order to safeguarding human health by establishing limits for air pollution due to NO_2_, among other air pollution variables, air quality estimations were conducted in Milan, Italy, from 2013 to 2016 [17]. The research presented in [17] was carried out by using machine learning and deep learning models aimed at obtaining a robust estimate of pollutants. Additionally, in [17] the following configurations were employed: a linear regressor, a multilayer perceptron with Bayesian regularization, a random forest regressor, and a long-short term memory network.

The concept of robustness used in [17] has different meanings. First, it was argued that the first linear model used in [17] can be seen as a standard ordinary least squares algorithm that reduces the influence of strong outliers in many cases. Furthermore, the authors of [17] suggested that the linear models used in the sense of their paper were used as a robust baseline.

Second, in [17] it was stated that due to the sampling procedure and ensemble learning of random forests, the latter provide solutions that are robust because they do not suffer from overfitting in the same way that simpler regression trees do.

Third, and finally, in [17] the F1-score was used as a robustness metric against unbalanced classes in a multi-class categorization problem, which was associated with the estimated time series produced by the trained models during an evaluation process of the regression estimates.

Seasonal variations in NO_x_ concentrations in Changchun (Jilin, China) were studied in [18]. In that paper, both monthly and daily average NO_x_ concentration variations were also studied. Additionally, in [18] the lineal dependence between NO_x_ and NO_2_, among other air pollutants in Changchun, was found. Moreover, in [18] the coefficient of divergence was employed to assess the differences between the spatial distribution of NO_2_ concentrations at several monitoring sites from 2016 to 2018.

In addition, the sensitivity of SO_2_, NO and NO_2_ concentrations to the relevant factors that had an effect on the air quality inside a 20% biodiesel air-conditioned bus was studied in [19]. The bus used in [19] was chosen from a fleet of Toledo (OH, USA) and continuous monitoring of the abovementioned pollutants were conducted with indoor temperature and indoor relative humidity as comfort parameters. The study time of [19] comprised the spring, summer, autumn, and winter seasons from April 2007 to March 2008. Additionally, in [19] a summary statistic of the seasons was presented and a linear dependence was detected among month, season and other variables.

Furthermore, in [19] the regression tree method was used to study the sensitivity for in-bus air pollutant concentrations (i.e., SO_2_, NO, and NO_2_), and the analysis of variance was used to determine the statistically significant variables. Lastly, in [19] the quantification of the relationship between the in-bus air pollutant concentrations and the statistically significant variables was carried out, and the dynamics of in-bus pollution was compared with atmospheric physics.

The papers shown above were focused on the statistical analysis of sets of NO_2_ concentration measurements that were carried out over long periods of time. In these papers, tools of parametric statistical inference or artificial intelligence or machine learning were used to estimate the central tendency of the data and, in some cases, to carry out its modeling. In this sense, the results obtained in these papers were satisfactory. Nevertheless, several of these papers lack of an exhaustive analysis of the central tendency of the data and, in general, of the dispersion of the data. Furthermore, they did not establish the similarities and differences that allow the construction of robust confidence intervals in which the central tendency of the data is found, with at least the 95% confidence level.

This is important, because robust statistical inference [13,14,15] allows significant conclusions to be drawn about the data, even when few data are available and without the need to eliminate outliers, which could be carriers of important information about the physical system that generated them.

Something worth mentioning is that in practically all the papers shown above [16,17,18,19] the authors faced the problem of missing data, and used different tools to fill in the gaps. However, these values are artificial and their sole purpose is to put an estimate of the true value that should go in those gaps. On the contrary, however, using robust statistics in the sense of [13,14,15] the researchers do not have to do the above, because the analysis does not require having a large amount of data to draw significant conclusions, nor does the data distribution need to be Gaussian or parametric. This last characteristic is also shared by nonparametric statistical inference [11,12].

The research presented in this paper is in total agreement with what was said in the previous paragraphs and can be seen as a continuation of the research presented in [4,5,20,21].

At this point, it is important to mentioned that an analysis of air pollution variables in Quito was also carried out in [8]. However, in the report presented in [8] the robust analysis of air pollution variables was not performed, and only the mean value and the maximum values of the set of observations were taken into account. Therefore, the research presented in this paper, together with the research presented in [4,5,20,21], could be used as reference material to study in a rigorous, comprehensive way the behavior of the NO_2_ concentration at Belisario station, from January 2008 to December 2019. Belisario is one of the most important air-quality monitoring stations of the Ministry of the Environment of Ecuador and belongs to Quito Metropolitan Atmospheric Monitoring Network [8].

The objectives of this paper are as follows:

(1) Group the NO_2_ concentration measurements, taken from 1 January 2008 to 31 December 2019 at Belisario station, in sets of variables that represent the years, months, days of the week, and hours of the day.
(2) Obtain estimates of the central tendency of the data and their dispersion, using classic, nonparametric, resampling, and robust methods.
(3) Categorize the data and find confidence intervals that allow quantifying the differences between categories.
(4) Find periodic behaviors in the variables.

Other research in which some robust statistics tools have also been used in some way are the following. In [22], robust linear regression models were used to reduce the influence of outliers in a least square fitting problem using M-estimation. The air pollution variables under study in [22] were the following: carbon monoxide (CO), carbon dioxide (CO_2_), nitrogen dioxide (NO_2_), ozone (O_3_), volatile organic compounds (VOC), and particulate matter (both PM_2.5_ and PM_10_).

Furthermore, some of the robust statistical methods used in the research presented in this paper were also used in [23] to compare results obtained in [23] for different lenses and calibration processes, in a three-dimensional reconstruction problem of archeological remains. Moreover, robust linear regression was also used in [24] to estimate black carbon concentration in two sites in Helsinki, Finland.

Finally, it is important to mention that nonparametric statistical methods have also been used to estimate the behavior of air pollution variables [25,26,27,28,29,30,31,32]. However, the results obtained using nonparametric methods are inferior to those obtained using robust methods, in the sense that robust methods are practically immune to the influence of extreme values. Therefore, robust methods generate confidence intervals that are narrower than those generated using nonparametric methods [5,20,21].

In this paper, in order to summarize the set of NO_2_ concentration measurements, summary statistics are provided in Section 2. In addition, the analysis of the sets of measurements using nonparametric methods is carried out in Section 3, and the robust analysis of these sets is carried out in Section 4. Section 5 of this paper provides a discussion of the results, and the conclusions of the paper are presented in Section 6.

## 2. Summary Statistics of 12 Years of NO_2_ Concentration Measurements at Belisario Station

According to [8], measurements were performed with Thermo Scientific Models 42C and 42i NO-NO_2_-NO_x_ analyzers [33,34]. These measuring instruments are used as measurement standards in many countries. For example, EPA has designated them as reference and equivalent methods [35]. The detailed explanation of the experimental conditions in which the measurements of air pollutants are carried out in Quito is given in [8].

In Quito, the monthly behavior of the NO_2_ concentration seems to repeat every year [8]. In addition, in [8] it is said that the lowest concentrations of NO_2_ occur in the summer and the highest in the months of March, October, and November. In this regard, it is important to mention that, as stated in [8], there could be reasons, such as rains, high speed winds and volcanic eruptions, that cause the NO_2_ concentration to rise or fall at certain times.

The data collection process in this research started on 1 January 2008 and ended on 31 December 2019, and the sampling period was equal to one hour [8,10]. Therefore, 12 years of NO_2_ concentration measurements corresponding to 105,193 data will be analyzed. However, since some data did not appear and others had negative values, the research was carried out with at least 96% of all possible data. In other words, less than 4% of the total of all possible data was lost. Furthermore, in accordance with [36], at least 75% percent of scheduled samples for each year were collected.

In this research, NO_2_ concentration measurements were divided into four families of sets. These families consisted of the sets that represent the 12 years under study, the sets that represent the 12 months of the year, the sets that represent 7 days a week, and the sets that represent the 24 h of the day in groups of 2 h. Additionally, the following variables were formed:

- $X_{k}$, k=1,⋯,12, stands for the set of samples collected in year 2007+k.
- $Y_{k}$, k=1,⋯,12, stands for the set of samples collected in the *k*-th month of the year.
- $Z_{k}$, k=1,⋯,7, stands for the set of samples collected on the *k*-th day of the week.
- $W_{k}$, k=1,⋯,12, stands for the set of samples collected at each of the 24 h of the day but with hours in groups of 2 h.

In this paper, since there were no problems related to the lack of data to carry out the analysis, the time instants in which no information was recorded were not taken into account, because one of the advantages of robust statistical analysis is that this type of analysis allows to draw significant conclusions even with a few samples. As can be seen, in the case study in this paper there was a huge number of samples. Therefore, taking into account the above, there were no data scarcity problems. Moreover, the variables under study were considered to be linearly independent, because the linear correlation values between the variables were very close to zero.

**Figure 1:**
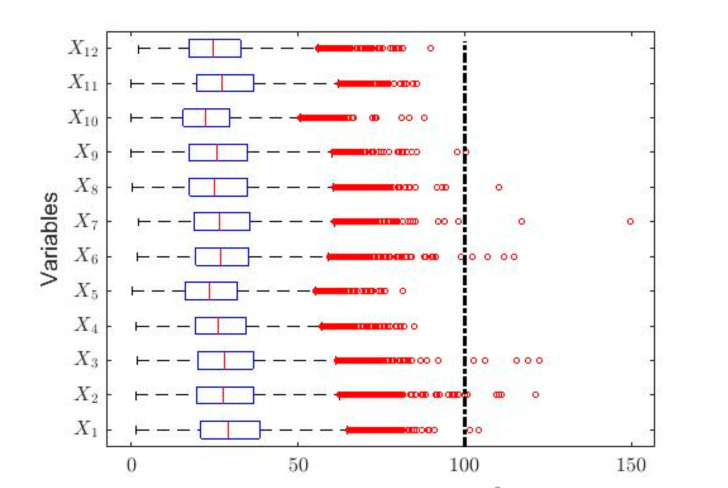
Box plots of the variables. The outliers are shown by using red circles and, according to QAQI [8], the dashed straight line indicates the separation between the desirable level of pollution (i.e., $\left[0,100\;\mu g/m^{3}\right)$ ) and the acceptable level of pollution (i.e., $\left[100\;\mu g/m^{3},200\;\mu g/m^{3}\right)$).

**Figure 2:**
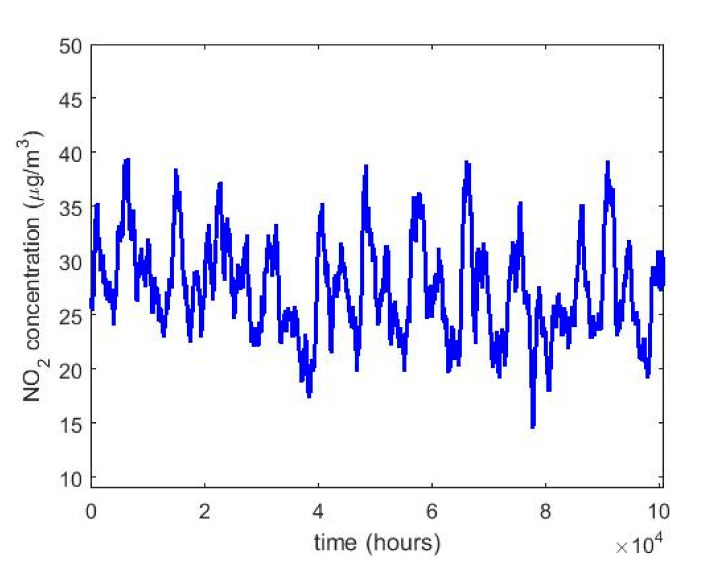
Moving average of the sequence consisting of the NO_2_ concentration measurements from 1 January 2008 to 31 December 2019.

**Figure 3:**
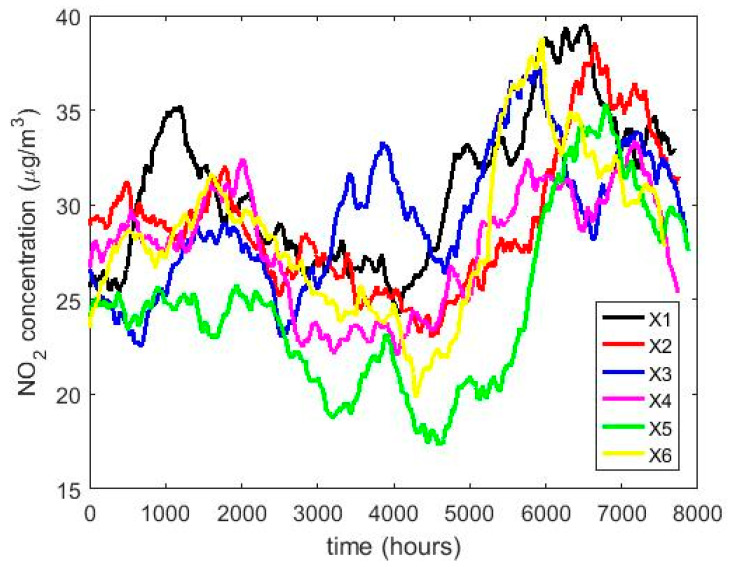
Moving average of the NO_2_ concentration measurements during the years from 2008 to 2013: $X_{1}\;\left(2008\right)$, $X_{2}\;\left(2009\right)$, $X_{3}\;\left(2010\right)$, $X_{4}\;\left(2011\right)$, $X_{5}\;\left(2012\right)$, and $X_{6}\;\left(2013\right)$.

**Figure 4:**
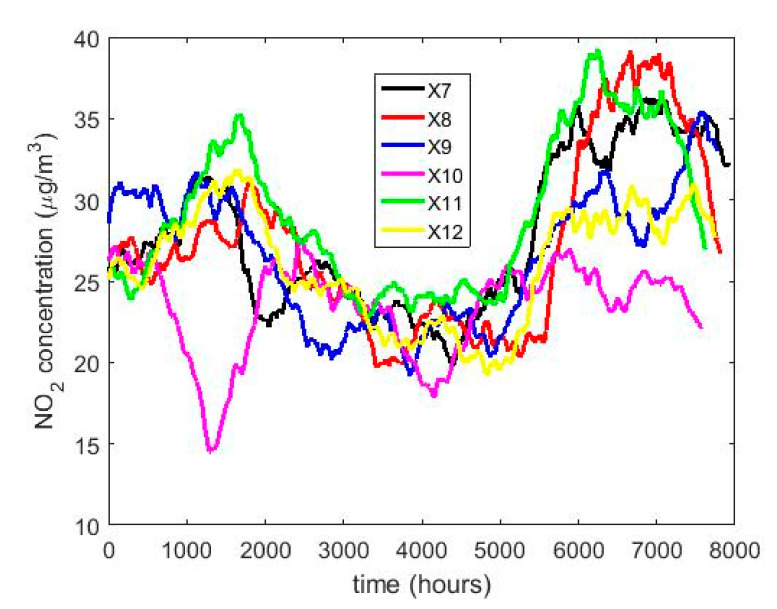
Moving average of the NO_2_ concentration measurements during the years from 2014 to 2019: $X_{7}\;\left(2014\right)$, $X_{8}\;\left(2015\right)$, $X_{9}\;\left(2016\right)$, $X_{10}\;\left(2017\right)$, $X_{11}\;\left(2018\right)$, and $X_{12}\;\left(2019\right)$.

In order to understand the characteristics of the NO_2_ concentration, making a preliminary analysis of the data, Table 1 shows a statistical summary of the samples collected. Additionally, Figure 1 shows the box plot diagrams of the NO_2_ concentration measurements by years, and the moving averages (MAs) of these measurements are shown in Figure 2, Figure 3 and Figure 4. Figure 2 shows the MA of the sequence consisting of all samples collected during the 12 years, Figure 3 shows the MA of the samples of the first six years, and Figure 4 shows the MA of the samples of the last six years. Time series studies use this type technique to analyze trends of variables [37,38].

In the box plots shown in Figure 1, a dashed straight line has been included indicating the separation between having a desirable level of pollution due to the NO_2_ concentration at Belisario station (i.e., $\left[0,100\;\mu g/m^{3}\right)$) and an acceptable level of pollution (i.e., $\left[100\;\mu g/m^{3},200\;\mu g/m^{3}\right)$). This classification, regarding the air pollution due to the NO_2_ concentration, is established in Quito by the Quito Air Quality Index (QAQI) [8].

The type of smoothing by using MA employed in this paper is the following: Given the sequence x of length k, find the average of the data set $x_{h},\;x_{h-1},\;\ldots ,\;x_{h-m+1}$ for each h≥m, where $x_{h}$ is the value of the sequence at the h position and m<k. This is done in order to make that each particular datum loses its individual influence, although this process makes that the researcher loses m−1 observations when analyzing the data. Here, the size of the MA was 720 due to the fact that this number is the number of data that there is in a 30-day month [5].

Table 1 shows that, for each of the variables under study, all the medians are less than the means. Furthermore, it is shown that the value of skewness is greater than zero and that all kurtosis values are greater than 3.4. In this sense, it should be noted that in the years 2013 and 2014 the kurtosis values are close to 5. Moreover, the values of the standard deviations are not small when compared with the values of the means, and Figure 1 shows that there are many outliers. All this indicates that the variables come from heavy-tailed distributions, or that their behavior may be due to the existence of a mix of distributions [14,39]. The aforementioned shows that these observations do not come from variables whose distribution is Gaussian [40].

In Figure 1, it can be seen that half of the years under study have observations that are above the desirable level of pollution [8]. Nevertheless, every year the NO_2_ concentration presents abnormally high observations. The latter again indicates that the variables under study do not come from Gaussian distributions, although the percentage of outliers does not exceed 2.1%.

Figure 2 shows that the NO_2_ concentration was stable during the 12 years of the study. In addition, it is observed that the maximum is reached in the third quarter of the year and the minimum in the second quarter. This is in accordance with what was said in [8]. Moreover, Figure 2, Figure 3 and Figure 4 show that once smoothing is performed, where each particular value loses importance with respect to the analysis in general, the values of the observations do not even reach half the maximum value of the desirable level of pollution. Therefore, exceeding the desirable level of pollution occurs at specific moments, which are never exceeded in a sustainable manner.

Due to the fact that there is a huge number of observations, in this paper classical statistical inference was tried to be used. However, as all the variables came from heavy-tailed distributions, several variable transformations [40] had to be carried out in order to make the variables fit a Gaussian distribution.

For the purpose of achieving smooth transformations around zero and to work with differentiable functions in the interval defined by the range of measured values, a transformation of the $T_{i}\;=\;\sqrt{X_{i}\;+\;1}$ type was used, where $X_{i}$ was the NO_2_ concentration in the *i*-th year, i =1,…, 12 (i.e., *X*_1_(2018),*X*_2_(2019), …, *X*_12_(2019))). One of the advantages of working with smooth transformations is that they share all the good properties that smooth functions have. Therefore, it is recommended to always look for simple transformations of the data to make them fit known distributions, starting with the Gaussian distribution [40].

**Figure 5:**
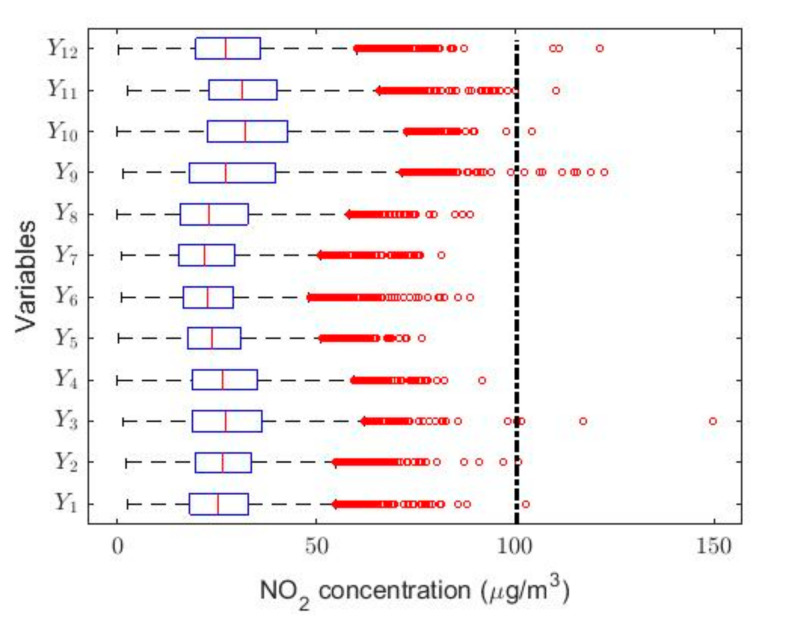
Box plot of data: $Y_{1}$ (January), $Y_{2}$ (February), $Y_{3}$ (March), $Y_{4}$ (April), $Y_{5}$ (May), $Y_{6}$ (June), $Y_{7}$ (July), $Y_{8}$ (August), $Y_{9}$ (September), $Y_{10}$ (October), $Y_{11}$ (November), and $Y_{12}$ (December).

**Figure 6:**
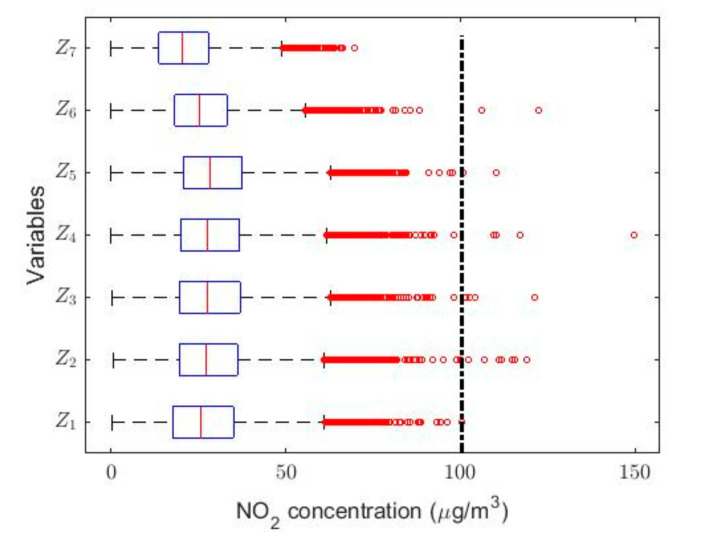
Box plot of data: $Z_{1}$ (Monday), $Z_{2}$ (Tuesday), $Z_{3}$ (Wednesday), $Z_{4}$ (Thursday), $Z_{5}$ (Friday), $Z_{6}$ (Saturday), and $Z_{7}$ (Sunday).

**Figure 7:**
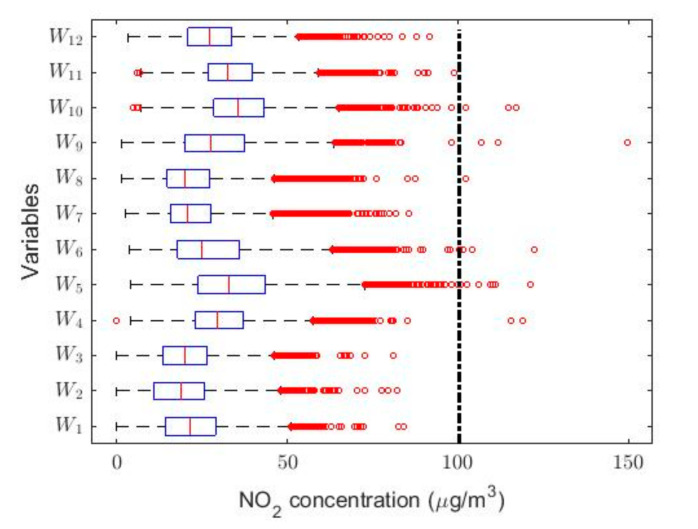
Box plot of data: $W_{1}$ (0:00–1:00), $W_{2}$ (2:00–3:00), $W_{3}$ (4:00–5:00), $W_{4}$ (6:00–7:00), $W_{5}$ (8:00–9:00), $W_{6}$ (10:00–11:00), $W_{7}$ (12:00–13:00), $W_{8}$ (14:00–15:00), $W_{9}$ (16:00–17:00), $W_{10}$ (18:00–19:00), $W_{11}$ (20:00–21:00), and $W_{12}$ (22:00–23:00).

**Figure 8:**
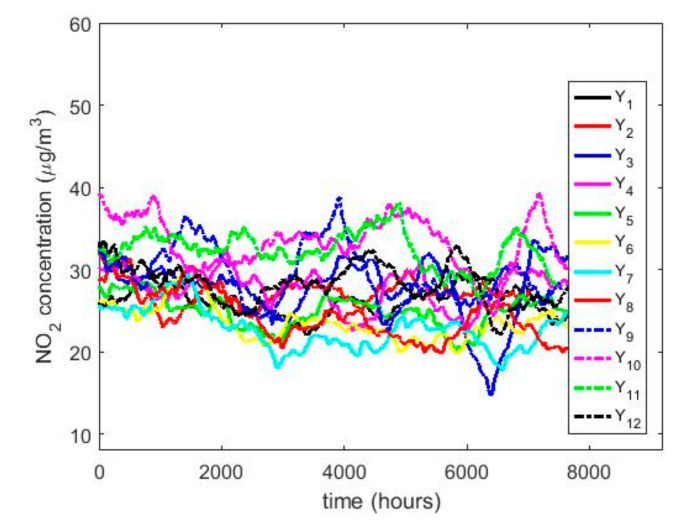
Moving average of the NO_2_ concentrations: $Y_{1}$ (January), $Y_{2}$ (February), $Y_{3}$ (March), $Y_{4}$ (April), $Y_{5}$ (May), $Y_{6}$ (June), $Y_{7}$ (July), $Y_{8}$ (August), $Y_{9}$ (September), $Y_{10}$ (October), $Y_{11}$ (November), and $Y_{12}$ (December).

**Figure 9:**
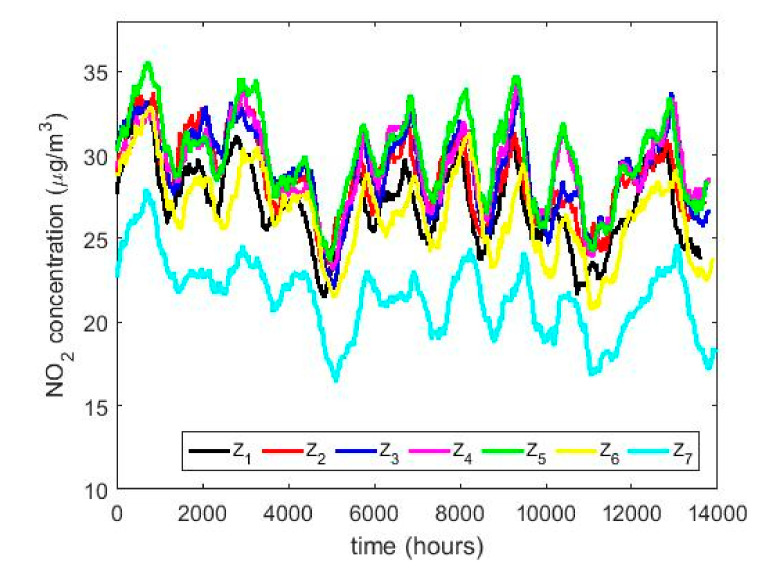
Moving average of the NO_2_ concentrations: $Z_{1}$ (Monday), $Z_{2}\;($Tuesday), $Z_{3}$ (Wednesday), $Z_{4}$ (Thursday), $Z_{5}$ (Friday), $Z_{6}$ (Saturday), and $Z_{7}$ (Sunday).

**Figure 10:**
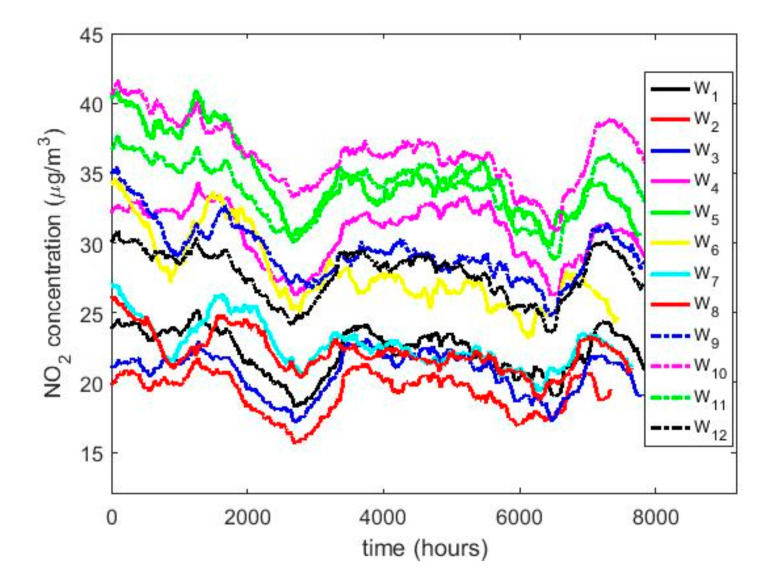
Moving average of the NO_2_ concentrations: $W_{1}$ (0:00–1:00), $W_{2}$ (2:00–3:00), $W_{3}$ (4:00–5:00), $W_{4}$ (6:00–7:00), $W_{5}$ (8:00–9:00), $W_{6}$ (10:00–11:00), $W_{7}$ (12:00–13:00), $W_{8}$ (14:00–15:00), $W_{9}$ (16:00–17:00), $W_{10}$ (18:00–19:00), $W_{11}$ (20:00–21:00), and $W_{12}$ (22:00–23:00).

In this research, with the variable change made, it was possible to adjust all the $T_{i}$ variables to normal random variables with *p*-values greater than 0.05, except for the years $2011\;\left(X_{4}\right)$ and $2015\;\left(X_{8}\right)$. However, the year 2011 could be adjusted to observations from a normal distribution by using the $T_{4}\;=\;\sqrt{X_{4}\;+\;4}$ transformation, and the year 2015 could also be adjusted to observations from a normal distribution by using the $T_{8}\;=\;\sqrt{X_{8}\;+\;0.5}$ transformation. In both cases, the adjustments were achieved with *p*-values greater than 0.05. This leads to the use of classical inference, although the results will be compared with the results provided by nonparametric statistics and robust statistics. In Figure 5, Figure 6, Figure 7, Figure 8, Figure 9 and Figure 10, the box plots and the smoothed observations are shown by using moving averages for the months of the year ($Y_{i},\;i=1,\ldots ,12$), for the days of the week ($Z_{i},\;i\;=\;1,\ldots ,7$), and for the hours of the day in groups of two hours ($W_{i},\;i\;=\;1,\ldots ,12$).

The box plots shown in Figure 5, Figure 6 and Figure 7 show that the variables taken into account have extreme observations on the right, which are generally close to each other, although occasionally some of the variables present very extreme observations. Therefore, the desirable NO_2_ concentration level is sometimes exceeded.

The behavior between the months of the year is very similar (see Figure 8) and it is also similar between the days of the week (see Figure 9), except on Sundays. On the other hand, when analyzing the behavior of the NO_2_ concentration during daylight hours (see Figure 7 and Figure 10), it is observed that the concentration seems to increase at certain hours in the morning (approximately at 8:00) and at certain hours of the night (approximately at 20:00).

To what has been said above, it must be added that there are no differences between the months, the days of the week, and the hours of the day with respect to the concentration of NO_2_ across the years. This is because the moving average graphs of each of the variables (see Figure 8, Figure 9 and Figure 10) seem to have behaviors close to periodicity, which is more noticeable when the measurement period is shorter (see Figure 9 and Figure 10).

Here, it is important to mention that this type of periodic behavior has also been observed in the analysis of other air pollution variables [21]. However, the perfect understanding of this behavior requires an in-depth analysis, because at first glance it is seen that the possible period of the signal depends on the time interval that is chosen for its representation. Therefore, if there is some kind of periodicity, it manifests itself in a modulated or variable way. Nevertheless, obtaining a mathematical model that captures the possible periodicity of this signal, together with amplitude, frequency and phase modulations, is beyond the scope of this paper and remains a future research topic.

In the same way that was done for the analysis by years, it was also attempted to make the variables that represent the months, days and hours of the day fit a Gaussian distribution. This process was conducted by carrying out several variable transformations. In the case under study, it was possible to fit the months to variables from Gaussian distributions with a *p*-value greater than 0.05 by using a transformation of the $T_{i}\;=\;\sqrt{Y_{i}\;+\;4}$ type. However, the variables representing the days of the week and hours of the day could not be fitted to any statistical distribution that were not a heavy-tailed distribution. Therefore, in this paper, for these last two types of variables, classical inference methods will be considered to a lesser extent than nonparametric and robust inference methods.

## 3. Analysis of NO_2_ Concentration Measurements Using Nonparametric Methods

This section is aimed at testing whether the variables have different medians by using nonparametric statistical methods. In this research, the variability of the measurements could be caused by some distinguishing features of the years, months, days, and hours of the day in which the measurements were taken. In addition, random causes could be generated by undesirable climatic conditions or by measurement noise of the measuring instrument.

In this paper, the variables under analysis have been considered to be linearly independent, because the linear correlation between them was very close to zero. Therefore, if there were some kind of linear dependency between the variables, that dependency would be very weak. Furthermore, it is important to mention that one of the objectives of this paper is to study whether the distributions of the variables are equal or not between them, and analyze the differences that may exist between the variables. Therefore, the study of the study of different types of nonlinear dependencies that may exist between variables is beyond the scope of the paper.

Here, the Wilcoxon rank sum test [11,12] was used to test whether the observations come from distributions with equal medians. This test was also satisfactorily used in [4,20,21,25,26]. The null hypothesis was $H_{0}:\;Median\;=\;M_{0}$, and the alternative hypothesis was $H_{1}:\;Median\;\neq \;M_{0}$. In short, if it is considered that both the null hypothesis is true and the observations are stable, then half of the observations will be less than $M_{0}$ and the others will be greater than $M_{0}$. For the analysis, the confidence level was $\left(1-\alpha \right)$, being α = 0.05 the significance level. Thus, the bilateral nonparametric confidence intervals for the median were calculated as in [4,20].

**Figure 11:**
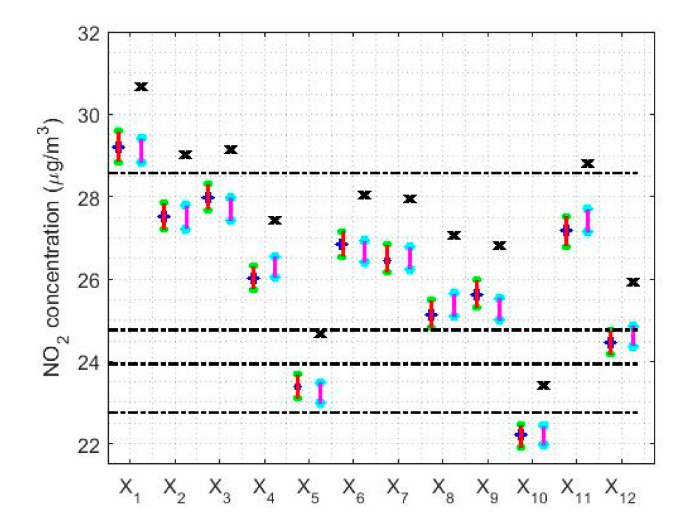
95% classic and nonparametric confidence intervals for the mean of the transformed data and the median of the untransformed data, respectively. The nonparametric confidence interval is shown to the left of each variable and the classical confidence interval to the right of each variable. The x above each confidence interval is the mean of the untransformed variable, and the horizontal dashed lines are used to separate the categories. $X_{1}$ ( 2008), $X_{2}$ (2009), $X_{3}$ (2010), $X_{4}$ (2011), $X_{5}$ (2012), $X_{6}$ (2013), $X_{7}$ (2014), $X_{8}$ (2015), $X_{9}$ (2016), $X_{10}$ (2017), $X_{11}$ (2018), and $X_{12}$ (2019).

With a confidence level equal to 95%, the limits of the confidence intervals for the median that were found in this paper are shown in Table 2. Additionally, a graphical representation of the confidence intervals for both the median and the transformed mean are shown in Figure 11. These confidence intervals are both classic and nonparametric. In Figure 11, the axis of the abscissa represents the variables (that is, the years) and the ordinate axis represents the NO_2_ concentration. In addition, in Figure 11, the nonparametric confidence interval centered on the median is represented to the left of each variable, while the classic confidence interval for the mean of the transformed data is represented on the right. Moreover, the x that is shown above each of the classic confidence intervals is the mean of the untransformed data of the displayed variable.

In Figure 11, all the means of the untransformed data are outside both the median-centered nonparametric confidence intervals and the classic confidence intervals for the transformed data, although the classic confidence intervals were built for the mean of the transformed data. Additionally, Figure 11 shows that there is a parallelism between the intervals found by the two methods (that is, the classic method and the nonparametric method) and that, in each variable, both confidence intervals contain the median of the data. However, it is important to mention that in the case of nonparametric intervals, the median is included in these intervals by construction of the intervals, which is not the case with the classic intervals. This fact corroborates that the data analysis carried out using nonparametric methods and whose distribution is not a normal distribution, produce satisfactory results in terms of location measurements.

Analyzing Figure 11, it can be seen how five categories have been established to classify the data, which are separated by horizontal dashed lines. The year 2008 is in the first category. The years 2009, 2010, 2011, 2013, 2014, 2015, 2016, and 2018 are in the second category. The year 2019 is in the third category. The year 2012 is in the fourth category and, lastly, the year 2017 is in the fifth category.

At this point, it is important to mention that all the categories are separated from each other by an NO_2_ concentration equal to a unit of measurement. Although, the third category has an amplitude of three units of measurement. However, there are differences in the widths of the measurement intervals. On the other hand, it is also verified again that the confidence intervals are at the desirable level of air pollution, according to QAQI [8]. Furthermore, in no case do the values of the confidence intervals come close to the acceptable level of air pollution due to the NO_2_ concentration, which shows that the desirable level of air pollution is exceeded in a circumstantial way.

**Figure 12:**
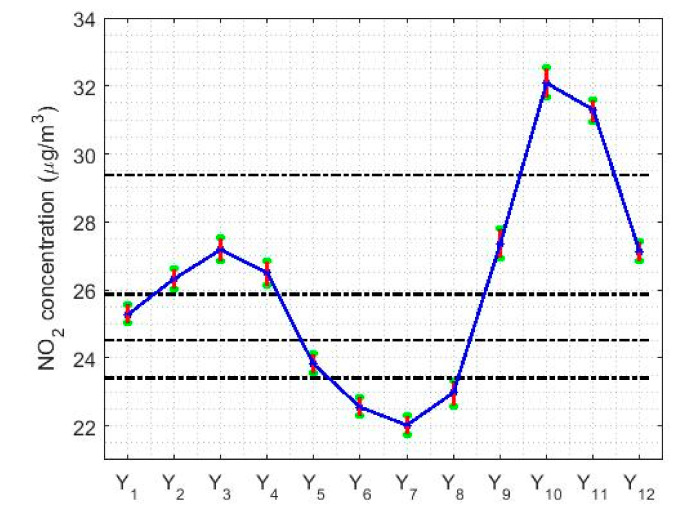
95% nonparametric confidence intervals for the medians: $Y_{1}$ (January), $Y_{2}$ (February), $Y_{3}$ (March), $Y_{4}$ (April), $Y_{5}$ (May), $Y_{6}$ (June), $Y_{7}$ (July), $Y_{8}$ (August), $Y_{9}$ (September), $Y_{10}$ (October), $Y_{11}$ (November), and $Y_{12}$ (December).

**Figure 13:**
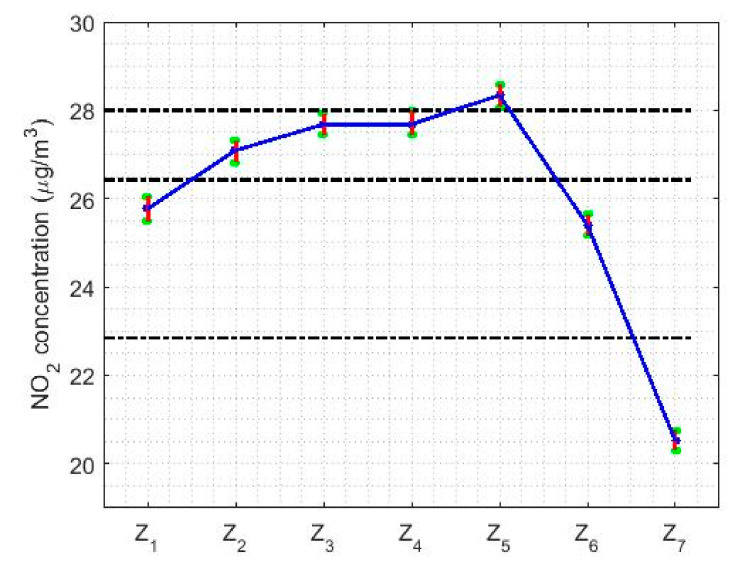
95% nonparametric confidence intervals for the medians: $Z_{1}$ (Monday), $Z_{2}$ (Tuesday), $Z_{3}$ (Wednesday), $Z_{4}$ (Thursday), $Z_{5}$ (Friday), $Z_{6}$ (Saturday), and $Z_{7}$ (Sunday).

**Figure 14:**
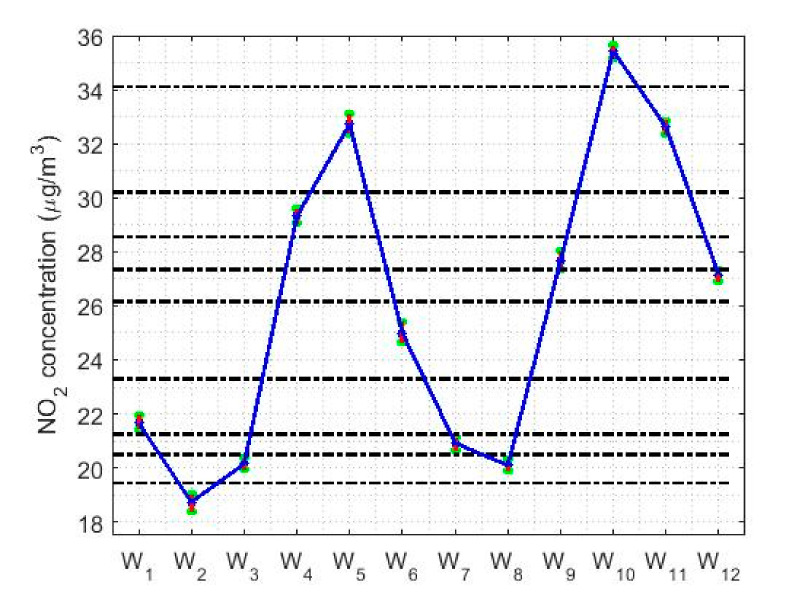
95% nonparametric confidence intervals for the medians: $W_{1}$ (0:00–1:00), $W_{2}$ (2:00–3:00), $W_{3}$ (4:00–5:00), $W_{4}$ (6:00–7:00), $W_{5}$ (8:00–9:00), $W_{6}$ (10:00–11:00), $W_{7}$ (12:00–13:00), $W_{8}$ (14:00–15:00), $W_{9}$ (16:00–17:00), $W_{10}$ (18:00–19:00), $W_{11}$ (20:00–21:00), and $W_{12}$ (22:00–23:00).

Next, Figure 12, Figure 13 and Figure 14 show the nonparametric confidence intervals for the medians of the variables that represent the months, days and hours of the day in groups of two hours.

As can be seen in Figure 12, five categories are established for the months using the nonparametric confidence intervals, which coincide with the results of the Wilcoxon rank sum test [11,12], taking into account low *p*-values. The NO_2_ concentration level, according to the months, seems to behave periodically across the years. The lowest concentration levels occur in summer, then it grows to the highest level obtained at the beginning of autumn, to lower and stabilize in winter and spring. At the end of spring, the decline in concentration begins. Although the variation is very small, the larger the median, the grater the width of the nonparametric confidence intervals. This feature has already been seen in other studies [20].

With respect to the analysis of the NO_2_ concentration by weeks, Figure 13 shows that this concentration decreases by 20% on weekends compared to working days, where there is a maximum on Fridays. Additionally, the NO_2_ concentration remains stable during the working days. The results obtained with these nonparametric confidence intervals are analogous to those obtained by using the Wilcoxon rank sum test. Four categories are established for the NO_2_ concentration: one formed on Sunday, another formed on Friday, a third formed on Tuesday, Wednesday and Thursday, and the last one formed on Monday and Saturday. This last category represents the transition from weekdays to weekends.

Finally, in the study of the hours of the day, Figure 14 shows that the NO_2_ concentration has two minimums, two maximums, and hours of transition between these extremes. The minimums occur at approximately 2:00 and 14:00 each day, while the maximums occur at 9:00 and 19:00. Furthermore, the increases and decreases in NO_2_ concentrations are very pronounced. These go from concentration values ranging from $18\;\mu g/m^{3}$ to $33\mu g/m^{3}$ or from $20\;\mu g/m^{3}$ to $36\;\mu g/m^{3}$, representing jumps in value almost twice the concentration. There are many categories similar to those obtained with the Wilcoxon rank sum test, but these can be summarized in five: one formed by each minimum, another formed by each maximum, and the rest of the categories are transition categories from one state to another.

## 4. Robust Analysis of the NO_2_ Concentration Measurements

Robust Statistics is concerned with carrying out the analysis using statistics that suffer little variation compared to samples that present observations that are far from the vast majority of the data [13,14,15]. Therefore, this paper is aimed at both obtaining measures of central tendency and scale that are insensitive to extreme observations and assessing which parameters can help characterize the variables that are under consideration.

Extreme observations have little influence on the behavior of robust estimators, because with these estimators the influence curves are bounded [41]. These curves are used to characterize robust estimators and are intended to measure the influence that one observation has on the others. Furthermore, in addition to the property of being bounded, the influence curves have other properties, such as continuity and differentiability.

In this paper, robust estimators are applied to the ordered sample [12] of $O_{1},\ldots ,\;O_{n}$, which is $O_{\left(1\right)}\;\leq \;O_{\left(2\right)}\;\leq \ldots \;\leq \;O_{\left(n\right)}$, where $O_{\left(1\right)}$ stands for the observation that has the smallest value, $O_{\left(2\right)}$ stands for the observation that has the second smallest value, and so on.

### 4.1. Estimators of Central Tendency and Scale

In this section, location statistics will be used to indicate around which values most of the data are grouped. In addition, these values will be used to obtain deductions that determine the center of the distributions. The statistics used in this section can be found in [13,14,15,42,43,44].

*L*-location estimators:

- Trimean $\left(TM\right)$ [15,42].
- α-trimmed mean $\left(T\left(\alpha \right)\right)$ [13,14,15].
- α-winsorized mean $\left(W\left(\alpha \right)\right)$ [13].

*M*-location estimators:

- Andrew’s wave $\left(T_{wa}\left(c\right)\right)$ [13,15].
- Biweight $\left(T_{bi}\left(c\right)\right)$ [13,14].

Scale estimators:

- Sample standard deviation $\left(s_{x}\right)$ [13,14].
- Mean absolute deviation $\left(MAD_{mean}\right)$ [13,14].
- Median absolute deviation (MAD) [13,14].
- One-half of the fourth-spread $\left(SRH\right)$ [13,43].
- Least median squares $\left(LMS\right)$ [14].
- Winsorized standard error ($s^{W}\left(\alpha \right)$) [15].
- Andrew’s wave ($s_{\omega a}\left(c\right)$) [13].
- Biweight ($S_{bi}\left(c\right)$) [13,14].
- Estimator based on a subrange $\left(C_{n}^{\alpha }\right)$ [44].

**Figure 15:**
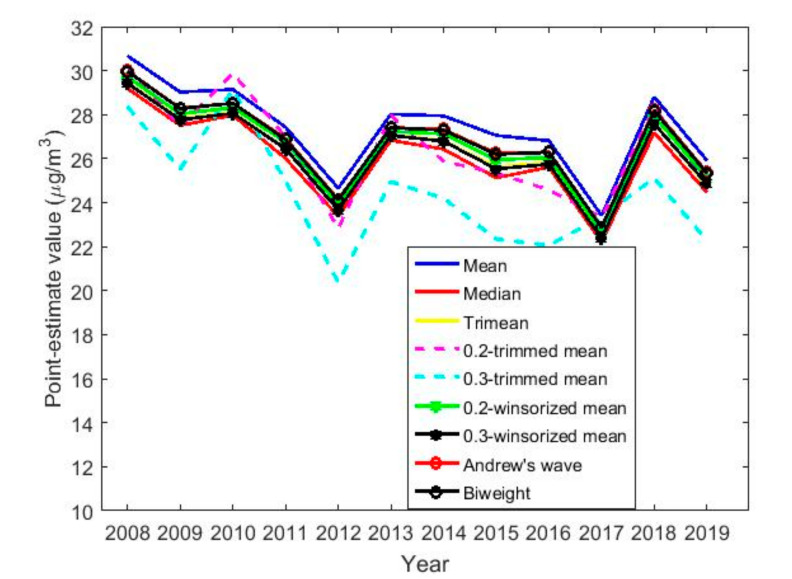
Graphical representation of the location estimates for the twelve years under study. Location estimators: mean, median, trimean, 0.2-trimmed mean, 0.3-trimmed mean, 0.2-winsorized mean, 0.3-winsorized mean, Andrew’s wave, and biweight.

**Figure 16:**
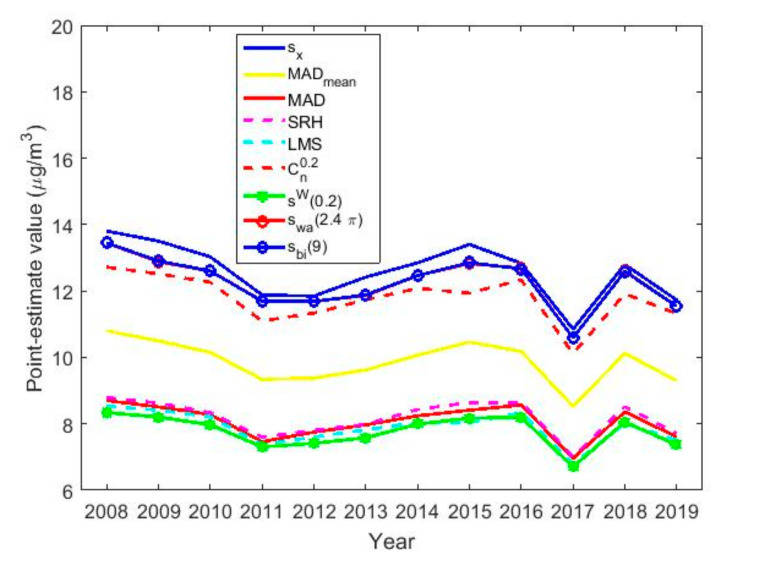
Graphical representation of the scale estimates for the twelve years under study. Scale estimators: sample standard deviation ($s_{x}$, mean absolute deviation ($MAD_{mean})$, median absolute deviation (MAD), one-half of the fourth-spread (SRH), least median squares (LMS), estimator based on a subrange ($C_{n}^{\alpha }$), winsorized standard error ($s^{W}\left(0.2\right)$), Andrew’s wave ($s_{\omega a}\left(2.4\pi \right)$), and biweight ($S_{bi}\left(c\right)$).

Table 3 shows the point estimates of location and the point estimates of scale are shown in Table 4. A graphical representation of the aforementioned location and scale estimators is shown in Figure 15 and Figure 16, respectively.

Figure 15 shows the location estimates, which indicate that NO2 concertation levels are very stable across the years. Furthermore, there is a maximum in 2008 and another in 2018. In addition, there is a minimum in 2012 and another in 2017, and the oscillation occurs between 20 $\left(\mu g/m^{3}\right)$ and 30 $\left(\mu g/m^{3}\right)$. Note that, in general, all measures of centralization for each variable fluctuate between the mean and 0.3-trimmed mean.

Figure 16 shows that all the scale estimators are very similar to each other, varying 3 $\left(\mu g/m^{3}\right)$ up or down. In addition, the behavior of all estimates is very uniform, growing and decreasing in the same periods. Moreover, it is observed that the standard deviation is the highest scale estimate and that the other scale estimates are bounded lower by the scale estimator 0.2-winsorized standard deviation.

At this point, it is important to note that these scale estimates are relatively high compared to the location estimates. The foregoing indicates that the variability in the NO_2_ concentration measurements is high. Furthermore, it indicates that although there are few outliers, there are many observations with high values, when these observations are compared with the center of the distribution. This had already been seen before when observing the amplitude of the box plot diagrams in Figure 1.

**Figure 17:**
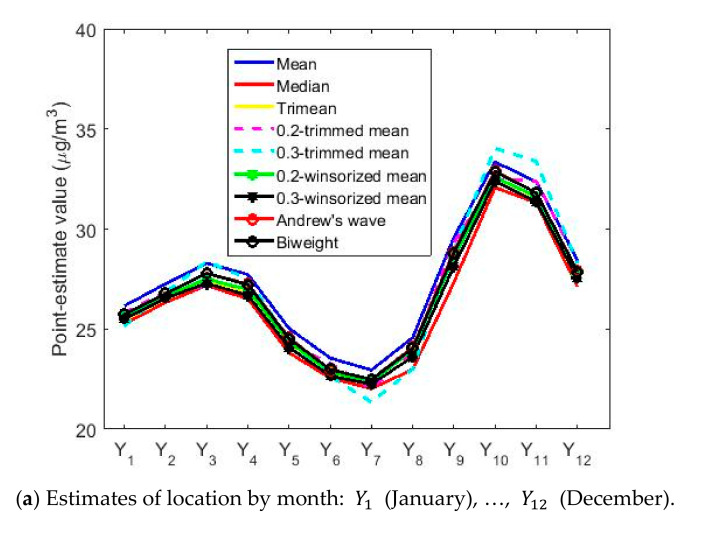

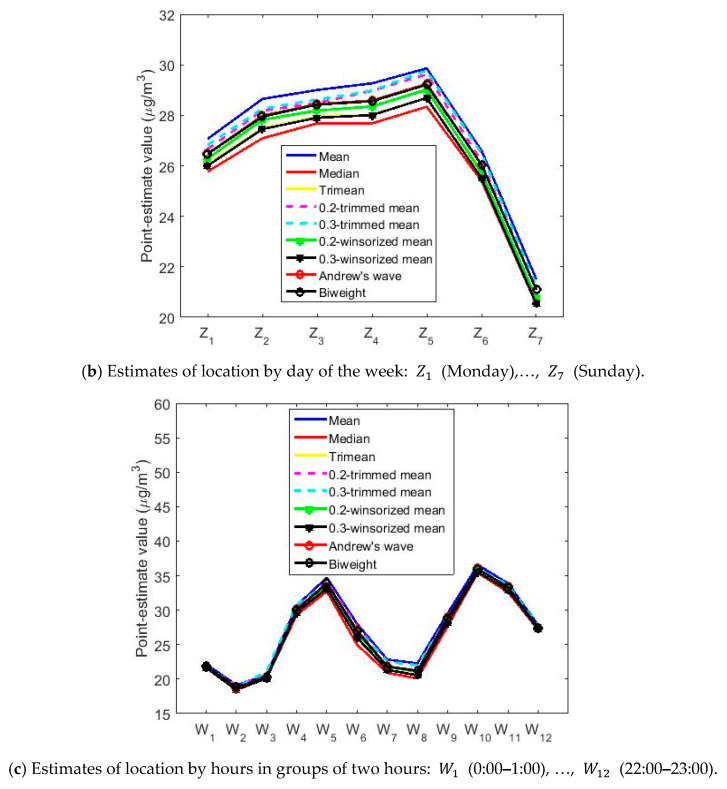
Estimates of location by month, day and hours.

**Figure 18:**
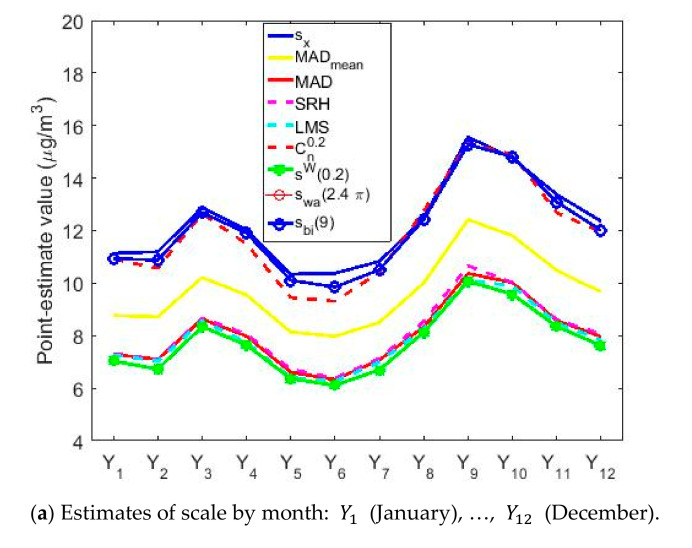

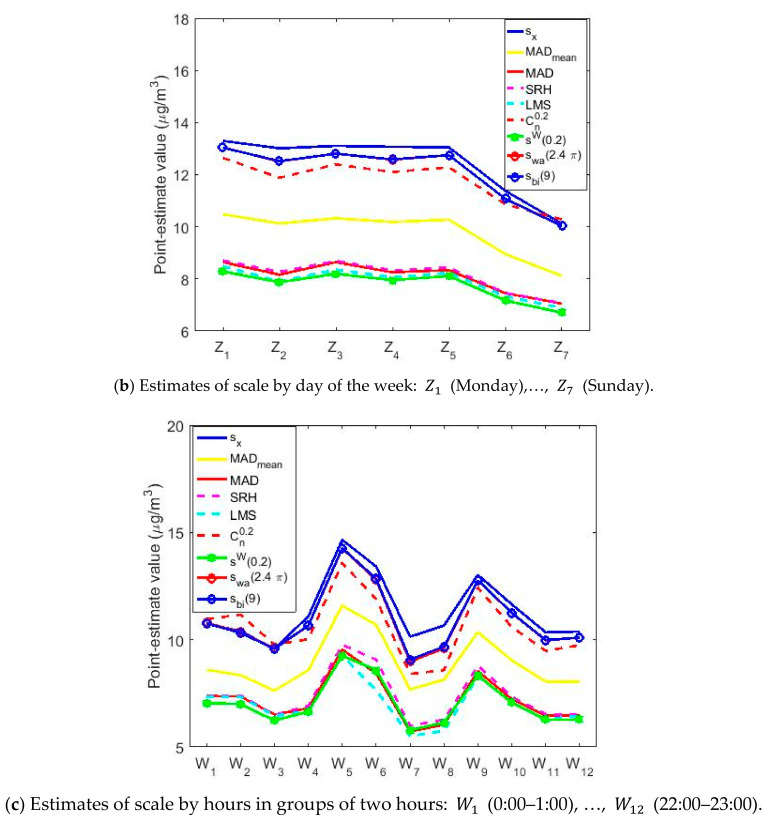
Estimates of scale by month, day and hours.

Figure 17 shows the location estimates by month, day and hour, where the variables are as follows: $Y_{1}$ (January), $Y_{2}$ (February), $Y_{3}$ (March), $Y_{4}$ (April), $Y_{5}$ (May), $Y_{6}$ (June), $Y_{7}$ (July), $Y_{8}$ (August), $Y_{9}$ (September), $Y_{10}$ (October), $Y_{11}$ (November), and $Y_{12}$ (December); $Z_{1}$ (Monday), $Z_{2}$ (Tuesday), $Z_{3}$ (Wednesday), $Z_{4}$ (Thursday), $Z_{5}$ (Friday), $Z_{6}$ (Saturday), and $Z_{7}$ (Sunday); an $W_{1}$ (0:00–1:00), $W_{2}$ (2:00–3:00), $W_{3}$ (4:00–5:00), $W_{4}$ (6:00–7:00), $W_{5}$ (8:00–9:00), $W_{6}$ (10:00–11:00), $W_{7}$ (12:00–13:00), $W_{8}$ (14:00–15:00), $W_{9}$ (16:00–17:00), $W_{10}$ (18:00–19:00), $W_{11}$ (20:00–21:00), and $W_{12}$ (22:00–23:00). In addition, for the abovementioned variables, the scale estimates are shown in Figure 18. 

In Figure 17, the estimates of location by months, days of the week and hours of the day in groups of two hours are very similar to each other, with the difference of 1 $\mu g/m^{3}$ from one type of estimator to another. Therefore, the difference between the estimators is negligible.

Figure 17a shows that the location estimates again reflect the same characteristics seen in the nonparametric estimates. In the analysis by months, the concentration of NO_2_ goes from having a minimum at approximately 22 $\mu g/m^{3}$, in the middle of summer, to having a maximum at the beginning of autumn, showing a growth of 50% in the concentration of NO_2_. Afterwards, there is a decrease in the concentration of NO_2_ tending towards 25 $\mu g/m^{3}$, where the NO_2_ concentration remains in a steady state until the arrival of the following summer.

In Figure 17b, the days of the week show two states, that on working days and that on weekends. On weekends, the NO_2_ concentration drops considerably compared to the value it reaches during working days. Lastly, Figure 17c shows that the hours of the day have two very pronounced minimums and maximums, in which the value of the NO_2_ concentration of the minimums is almost doubled.

Comparing Figure 17 and Figure 18, it is observed that the scale estimates are very high with respect to the location estimates, which suggests high variability in the data, although with few outliers. In addition, Figure 18 shows that the scale estimates are grouped into three steps: (1) the step formed by the highest estimates, $s_{x}$, $s_{wa}\left(2.4\pi \right)$, $s_{bi}\left(9\right)$ and $C_{n}^{0.2}$; (2) the step formed by $MAD_{mean}$; and (3) the step formed by lowest estimates, LMS, SRH, MAD and 0.2-winsorized standard deviation.

Figure 18 also shows that the variables representing the months, days and hours seem to follow the same pattern. Specifically, these variables increase and decrease at the same times, for the group that represents the months (see Figure 18a), the one that represents the days of the week (see Figure 18b) and the one that represents the hours of the day (see Figure 18c).

Finally, it is observed that there is a concordance between location and scale estimates. Specifically, the increase in the NO_2_ concentration leads to an increase in its variability.

### 4.2. Confidence Intervals

Following the methodology used in [5,20,21], in this section confidence intervals will be constructed to classify the variables under study, categorize said variables, and establish similarities and differences between these variables that bring out possible patterns of behavior of the concentration of NO_2_ at Belisario station. In addition, the location and scale estimators presented in Section 4.1 were used to build the following confidence intervals.

- $\left(\bar{X},s_{x}\right)$, where $\bar{X}$ stands for the mean.
- $\left(M_{e},\;MAD\right)$, where $M_{e}$ stands for the median.
- $\left(M_{e},\;IQR\right)$, where IQR stands for the interquartile range.
- $\left(T\left(\alpha \right),s^{W}\left(\alpha \right)\right)$.
- $\left(T_{wa}\left(c\right),s_{wa}\left(c\right)\right)$.
- $\left(T_{bi}\left(c\right),s_{bi}\left(c\right)\right)$.

Furthermore, a bootstrap method was used to build confidence intervals [15,20,21]. With all the above, nine confidence intervals were built for all variables: one classic confidence interval, one classic confidence interval based on data inversion of the transformed data using the function $\sqrt{X_{i}+1}$, being $X_{i}$ the *i*-th variable to be transformed, one confidence interval based on a bootstrap method, one nonparametric confidence interval, and five robust confidence intervals.

**Figure 19:**
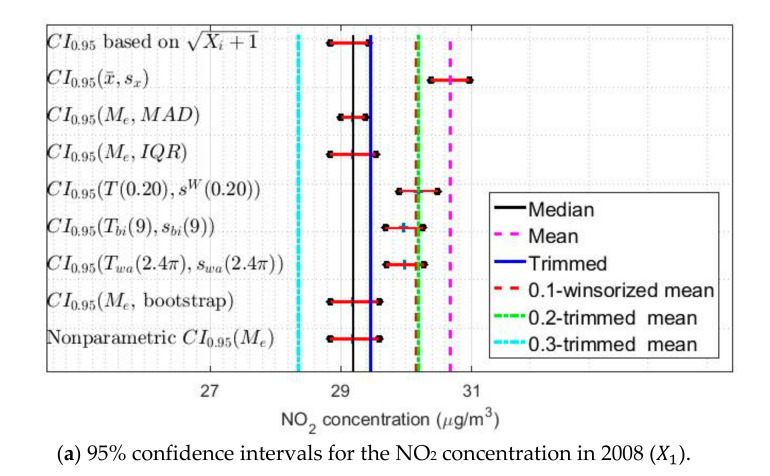

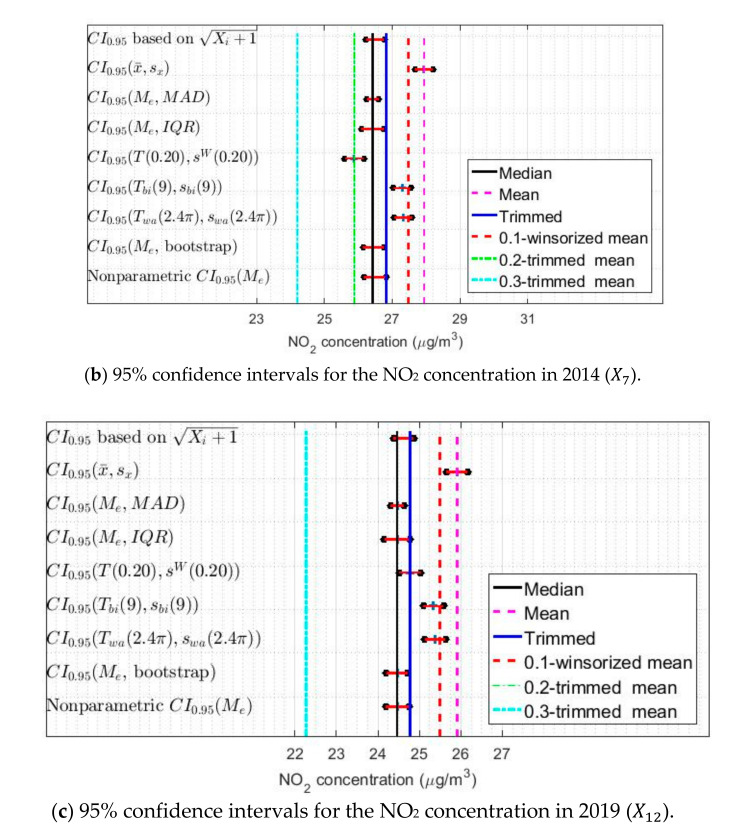
95% confidence intervals ($CI_{0.95}$): classic, classic based on data inversion of transformed data, robust, boostrap, and nonparametric confidence intervals.

Figure 19 shows the confidence intervals built for the years 2008, 2014 and 2019. Showing the confidence intervals for more variables does not provide significant information, because these intervals present the same characteristics for all the variables. 

Specifically, the classic confidence intervals are those that are the most displaced towards the highest values, while the confidence intervals based on the Andrew’s wave and the biweight are analogous in all variables. Furthermore, these two types of intervals cover lower values than those mentioned above, with a difference equal to $1\;\mu g/m^{3}$. The rest of the confidence intervals cover the median of the data and differ from the first ones by $1\;\mu g/m^{3}$. Therefore, the difference between the highest estimates and the lowest estimates is equal to $2\;\mu g/m^{3}$, which is practically negligible.

Taking into account all the above, the pairs of estimators $\left(T\left(\alpha \right),s^{W}\left(\alpha \right)\right)$ and $\left(T_{bi}\left(c\right),s_{bi}\left(c\right)\right)$, for α = 0.2 and c = 9, will be used to carry out the comparison of the variables. The reasons that justify having made this decision are the following.

First, the classical confidence intervals are based on the underlying distribution of the data being approximately normal, which is not true in this research.

Second, the results obtained with the estimators $\left(M_{e},MAD\right)$, $\left(M_{e},IQR\right)$ and bootstrap are analogous to those obtained by the nonparametric estimators. Although, it must be said that the pair of estimators $\left(M_{e},MAD\right)$ allows us to better observe the differences in the grouping of variables. Therefore, the possibility of carrying out the grouping of similar behaviors in the NO_2_ concentration by years has been lost.

Third, and finally, as the results obtained with the Andrew’s wave are similar to those obtained with the biweight, any one of these two estimators can be chosen to perform the analysis.

**Figure 20:**
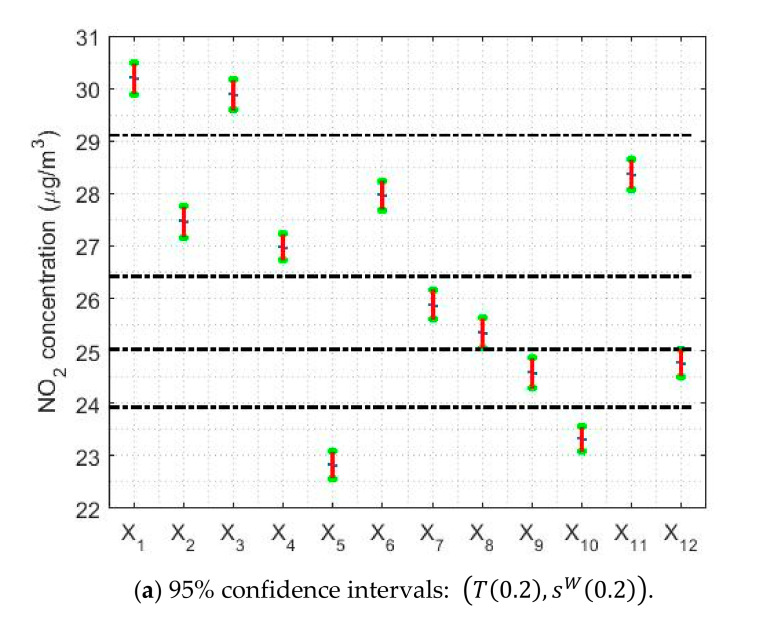

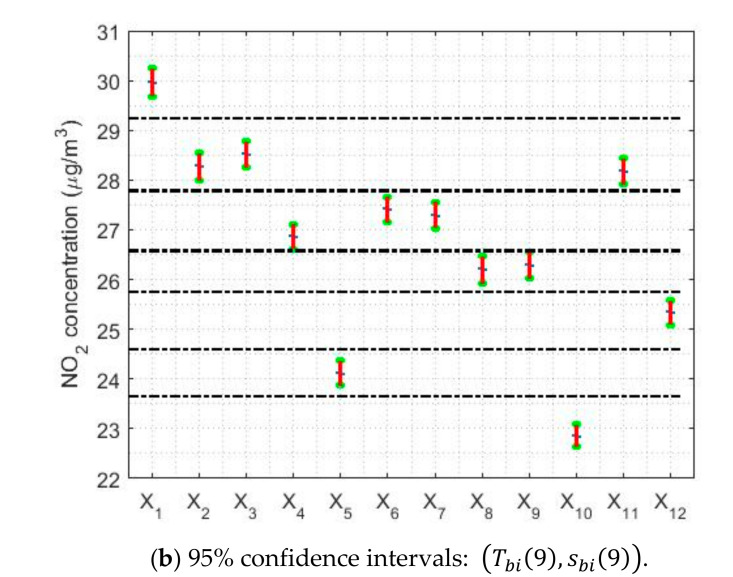
95% confidence intervals by using the pairs of estimators (*T*(0.2),*s^W^* (0.2)) and (*T_bi_* (9),*s_bi_* (9)).

Table 5 shows the limits and lengths of the 95% confidence intervals for $\left(T\left(0.2\right),s^{W}\left(0.2\right)\right)$) and $\left(T_{bi}\left(9\right),s_{bi}\left(9\right)\right)$. In addition, a graphical representation of the confidence intervals is shown in Figure 20. In this figure, horizontal dashed lines have been included to classify the variables. Note that this was done previously when the classification of the medians provided by the Wilcoxon rank sum test was performed in Section 3, when building the nonparametric confidence intervals. When looking at Figure 20, it is important to mention that with the estimators (*T*(0.2),*s^W^* (0.2)) and (*T_bi_* (9),*s_bi_* (9)) the classifications of the variables are more refined than those obtained by the nonparametric estimators. Nevertheless, it must be realized that the differences between one another do not reach 1 $\left(\mu g/m^{3}\right)$, which represents a very insignificant difference.

Regarding the amplitudes of the confidence intervals, Figure 20 shows that the confidence intervals found with the pair of estimators $\left(T_{bi}\left(9\right),s_{bi}\left(9\right)\right)$ are narrower than the confidence intervals found with the pair of estimators $\left(T\left(0.2\right),s^{W}\left(0.2\right)\right)$. Specifically, the width of the confidence intervals found with $\left(T_{bi}\left(9\right),s_{bi}\left(9\right)\right)$ is between 2.8% and 7.2% narrower than the width of the intervals found with $\left(T\left(0.2\right),s^{W}\left(0.2\right)\right)$. Furthermore, it is important to mention that the amplitudes of these intervals evolve in parallel to the median of the data.

**Figure 21:**
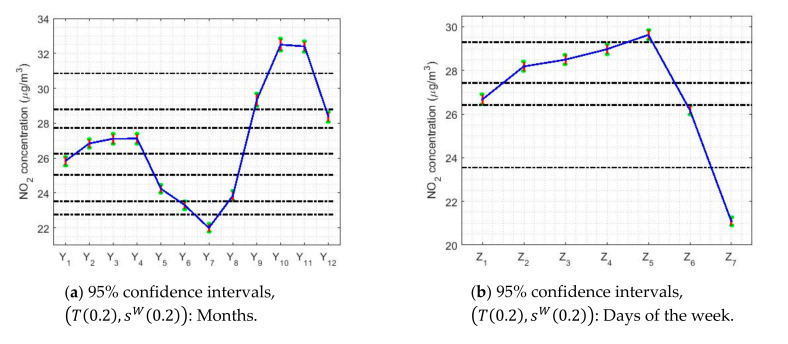

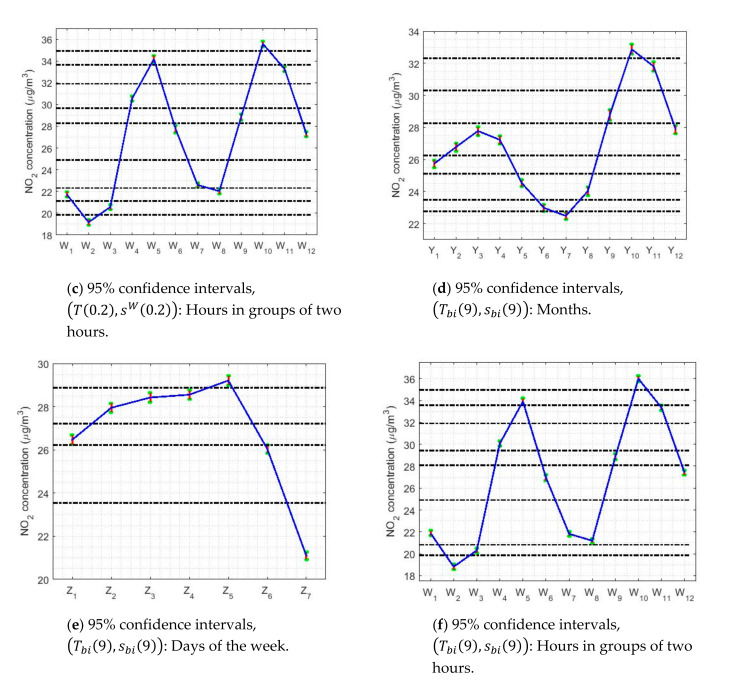
95% confidence intervals, (*T*(0.2),*s^W^* (0.2)) and (*T_bi_* (9),*s_bi_* (9)), by month (*Y*_1_ = January, …, *Y*_12_ = December), day of the week (*Z*_1_ = Monday, …, *Z*_7_ = Sunday), and every two hours (*W*_1_ = 0:00–1:00, …, *W*_12_ = 22:00–23:00).

Figure 21 shows the confidence intervals based on $\left(T\left(0.2\right),s^{W}\left(0.2\right)\right)$ and $\left(T_{bi}\left(9\right),s_{bi}\left(9\right)\right)$ for the analysis by months, days and hours. In Figure 21a,d, it is observed that the lowest NO_2_ concentration values are reached in the month of July, with values that are approximately equal to $20\mu g/m^{3}$. 

From that moment, the NO_2_ concentration rises until reaching to its maximum value, which is approximately equal to $33\;\mu g/m^{3}$ and which is reached in October. In other words, in the summer months the NO_2_ concentration drops to a little more than half its maximum value. Furthermore, the drop in NO_2_ concentration levels appears to have two steps. The first of these steps is reached at the end of the year, at which point the NO_2_ concentration stabilizes and remains stable until April. Then, the NO_2_ concentration drops again until it reaches its minimum value in July. These results are similar to those obtained in Figure 12. Furthermore, the amplitudes of the confidence intervals seem, in general, to be smaller than for the analysis by years. Likewise, there is also the effect that the higher the median value, the greater the width of the intervals.

Regarding the analysis by days of the week (see Figure 21b,e), it can be said that this is in every way analogous to that obtained with nonparametric estimators (see Figure 13). On weekends, the NO_2_ concentration reaches the minimum, reducing to 25% of the value reached on weekdays. In addition, there are three categories: one for Sundays, another for weekdays, and then the transition categories. Although, it should be noted that on Fridays the highest NO_2_ concentration value of the entire week is reached.

Finally, with respect to the analysis by hours of the day (see Figure 21c,f), it is important to say that the results are also very similar to those found with nonparametric estimators (see Figure 14). For both types of confidence intervals, the NO_2_ concentration reaches maximum values at approximately 9:00 and 19:00. That is, the maximum is reached in the first hours of the beginning of the working day and at the end of the working day. In addition, the concentration of NO_2_ suffers very pronounced falls, reaching two relative minimums: one at approximately 2:00 and the other at approximately 14:00, the minimum reached at 2:00 being the deepest. Among the abovementioned minimums and maximums, there are only transitioning levels, because there is no any value at which the NO_2_ concentration remains stable for several hours.

## 5. Discussion

From a statistical summary of the data it could be observed that the vast majority of the observations are at a desirable level of air pollution, due to the levels of NO_2_ concentrations at the Belisario air-quality monitoring station [9]. Furthermore, those observations that were not at a desirable level of pollution were few extreme values that were at an acceptable level of air pollution. The criteria used in Quito to establish desirable and acceptable levels of air pollution are defined by QAQI [8]. Although, it is worth mentioning that each urban city in the world sets its own criteria. Therefore, it could happen that in other cities of the world, the desirable level of air pollution is only used to say that the level of air pollution due to the concentration of air pollution variables is 0 and that the rest of the levels range from being not harmful to humans to the danger of death.

To the aforementioned, it must be added that, after a preliminary analysis of the data, it was observed that the samples came from heavy-tailed distributions. But, it was possible to discover transformations that allowed to carry out the transformation of the original variables into other variables from Gaussian distributions. However, this was only possible for some types of variables. Therefore, it became necessary to combine various types of statistical analysis to be able to explain in a precise, exhaustive and comprehensive way the behavior of the NO_2_ concentration at the Belisario air-quality monitoring station.

Within the part of the research aimed at carrying out the descriptive analysis of the data, the original data were also smoothed to try to reduce the influence that each datum has on the rest of the data. This process revealed that observations show low air pollution values, below half the desirable level of pollution. Therefore, exceeding this level of air pollution is something very specific and does not sustain over time. Thus, at first glance, this confirms that the level of air pollution at Belisario station is not harmful to humans. This result is in agreement with what was said in [8].

In general, this type of comprehensive preliminary analysis of measurements of NO_2_ concentrations in urban cities is not very common. For example, in [16,17,18,19] researchers tend to eliminate outliers following certain criteria and then use artificial intelligence or machine learning tools to carry out data analysis. In addition, after the data cleaning process, including the elimination of extreme values, researchers tend to look for linear dependencies between the variables in order to apply linear analysis tools and interpret the data in this way.

However, unlike what was said previously, in this research it was decided to analyze the data taking into account the contribution of extreme values, because these values are the response of the dynamic system under study to certain types of inputs. Therefore, in this research the extreme values were considered as carriers of useful information and allowed to justify the robust analysis of the data.

Nonparametric statistical inference tools have also been used in the analysis of air pollution variables [25,26,27,28,29,30,31,32]. This has been important, because if the data do not follow parametric distributions, then classical statistical inference is unfounded, which is the case of study in this research. In this sense, in [25,26,27,28,29,30,31,32] nonparametric analysis tools have been used to study several variables of air pollution. Nevertheless, nonparametric analysis is much more sensitive to the influence of extreme values than robust analysis. But it also provides relevant information that allows to classify data and determine similarities and differences between them, which also allows the researcher to categorize variables. All of this has been done in this research and has paved the way for applying robust data analysis tools.

In this paper, nonparametric and robust statistical hypothesis testing and nonparametric and robust confidence intervals were also used to compare the results with the results obtained using classical techniques. Furthermore, this was used to justify why not all the variables supported the analyzes using classical techniques. In addition, using the Wilcoxon rank sum test, it was verified that the variables could be grouped into different groups.

Here, the results obtained by using nonparametric confidence intervals for the median were very similar to those obtained by using the classical methods applied to the transformed observations. In short, for the analysis by years, five strata were established: four formed by a single year, 2008, 2012, 2017 and 2019, and another formed by the other years. Each stratum differs from the others by $1\;\mu g/m^{3}$.

The classes for the months were five, but could be reduced to four: one class for the maximum, $Y_{10}$ (October), another class for the minimum, $Y_{7}$ (July), the third class for the end of the year and the first quarter of each year, and the last class formed by moments of transition between the three abovementioned classes.

On the other hand, the days of the week are grouped as follows. First, on the central three days of the work week (that is, Tuesday, Wednesday, and Thursday). Second, on Fridays, this part being the highest in terms of NO_2_ concentration levels. Third, on Sundays, this part being the one with the lowest concentration values. Fourth, and finally, on Saturdays and Mondays, which are part of the transition from some levels to other levels. Furthermore, the value of the reduction of the NO_2_ concentration levels from Friday to Sunday is approximately equal to one third of the NO_2_ concentration levels on Friday.

Regarding the analysis by hours, several groupings were also obtained. These groups have a maximum at 9:00 and another at 19:00, and two minimums, one at the end of business hours and another in the early hours of the morning.

Next, as was done in [5,20,21], different location and scale statistics were found, which were used to build robust confidence intervals. At this point, it is worth mentioning that all location estimates were bounded by the median and the 0.2-trimmed mean. In addition, it is clearly observed that the concentration of NO_2_ has all its values in the range of desirable values, decreases in the years 2012, 2017 and 2019, and is higher in 2008. But, the difference between the greatest concentration value and the smallest value on each curve representing the location estimates by year is less than $9\;\mu g/m^{3}$.

Regarding the scale statistics used, these were the same as those used in [5,20,21]. In addition, for the design of the families of estimators, values that are mentioned in the specialized literature as suitable values were chosen.

All the scale estimates are in one band, where the standard deviation is well above all of them and the rest of the estimates have the least median of squares as the lower bound. Here, there is a parallel between the location and scale estimates, in the sense that the rise (respectively decrease) in the value of the location estimate produces a rise (respectively decrease) in the scale estimates. All this leads to the conclusion that extreme observations, both in quantity and value, are the observations that determine the values of the location and scale estimates.

After analyzing the robust confidence intervals, it was concluded that the most appropriate pairs of estimators for the analysis based on confidence intervals were $\left(T\left(0.2\right),s^{W}\left(0.2\right)\right)$ and $\left(T_{bi}\left(9\right),s_{bi}\left(9\right)\right)$. The classifications of the variables made with these pairs of estimators were very similar to those obtained with the nonparametric estimators and with the classical methods applied to the transformed variables, in the case of the years, but with small differences.

On the other hand, in the analysis of the variables by months, days and hours, it is observed that there is a possible periodicity. Specifically, the analysis by months shows notable rises at the beginning of autumn and falls in April and July. Furthermore, stability is observed in the first quarter and in the last months of each year. In the analysis by days of the week, there is a notable difference between the NO_2_ concentration on weekdays, with a higher concentration on Fridays, and on weekends. Finally, in the hourly analysis, maximum values are also seen at 9:00 and 19:00. The minimums are found in the early afternoon and early morning, while the rest of the categories are transition categories from one level to another, without periods of stable concentrations.

With all the above, groupings of variables were obtained by comparing the results obtained by years, months, days of the week, and hours. In addition, the differences between categories of variables were found and these differences were quantified using both robust and nonrobust confidence intervals.

A comprehensive study of NO_2_ concentration trends at Belisario station has been carried out in this paper. The results of this study showed that the NO_2_ concentration at Belisario station is stable when is analyzed by years, it is highly variable when is analyzed by months and hours of the day, and it is slightly changeable when is analyzed by days of the week.

Regarding the behavior of the NO_2_ concentration shown in this paper, it is important to say that this behavior bears some similarity to that of other pollution variables in urban cities. For example, in [21] maximums and minimums were also observed in the CO concentration in hours of the day close to those shown in the present paper. The aforementioned is important, because the study carried out in [21] was also carried out at Belisario station during the same time interval that was taken into account in the present paper. Another example that shows the existence of maximums and minimums at different times of the day in the behavior of the concentration of pollutants in urban cities, is the one shown in [24]. Specifically, in [24] it is observed how the concentration of black carbon varies throughout the hours of the day in two air quality monitoring stations located at Mäkelänkatu and Kumpula in Helsinki.

The behavior of the concentration of pollutants observed both in this paper and in the research carried out in [21,24], among others, shows that anthropogenic emissions play a fundamental role in the concentration levels of pollutants in urban cities.

Before finishing this section, it is important to say that the abovementioned possible periodic behavior has also been observed in the behavior of other air pollution variables [21]. However, data analysis showed that this behavior was only observed when the data were analyzed for certain time scales. Therefore, a transient phenomenon or a variable frequency signal could be observed, among other things. Here, this possible periodic behavior was shown by using robust confidence intervals. Additionally, the values of the possible periodic wave were categorized and the differences between categories were found with the measurement precision provided by robust statistical methods. All this constitutes another contribution of this research. Nevertheless, the analysis of the possible periodicity of this signal is beyond the scope of this paper, but it remains as a future research topic.

## 6. Conclusions

The objective of this paper was to carry out the robust analysis of the behavior of the NO_2_ concentration at the Belisario air-quality monitoring station, Quito, Ecuador from 1 January 2008 to 31 December 2019. Here, the NO_2_ concentration was decomposed into variables whose behavior was analyzed by years, months, days and hours of the day. Furthermore, after verifying that no set of separate variables came from the same distribution, the differences between parameters that characterized these variables were determined.

This is the first time that an exhaustive statistical analysis of the NO_2_ concentration at Belisario station has been carried out. In the report presented in [8], the concentration of several variables of air pollution in Quito was analyzed in a general way, but a robust statistical analysis was not carried out. Specifically, the analysis carried out in [8] only took into account the mean and maximum values of the concentration of the air pollution variables. Therefore, the research presented in this paper could serve as a reference material to comprehensively analyze the NO_2_ concentration at Belisario station in the last 12 years. This highlights some of the possible uses of the results of this research.

The results of the study conducted here robustly proved that the NO_2_ concentration levels at Belisario station are not harmful to humans. In addition, it was also shown that the behavior of this concentration tends to be stable across the years, changes slightly during the days of the week, and varies greatly when analyzed by months and hours of the day.

In the report presented in [8], the main sources of air pollution in Quito are mentioned, highlighting among them the means of transport, large traffic jams, and all the industries that are located in the capital and its surroundings that use bunker and fuel oil. Additionally, since Quito is a long, narrow city, there are many traffic jams in the city center when traveling from one end of the city to the other. To all this it must be added that the city center is located on the slopes of the Pichincha volcano, and volcanic eruptions are air pollution sources [8].

What has been said in the previous paragraph highlights the need to improve the urban dynamics of the city, in order to contribute to the reduction of NO_2_ concentration levels across the city. In this sense, the authors of this research suggest the need to carry out the set of proposals made by themselves in [4,21]. For example, it is important to improve the quality of the main means of transport that are used in the city, build more green areas that serve as effective filters for air pollution, and establish air pollution criteria that are adaptive, being more severe in the regions of the city where there is a greater concentration of people. For more information on things that can be done to improve air quality in urban cities, it is recommended to see [4,21], for the case of Quito, and other scientific publications dedicated only to this topic worldwide.

Finally, depending on the time interval used to represent the data, a possible periodicity in the NO_2_ concentration measurements at Belisario station was observed. However, the in-depth mathematical modeling of this signal is a complex issue that falls outside the scope of this research, but remains pending to be performed in a future research of the authors.

## Figures and Tables

**Table 1:** Summary statistics of the NO_2_ concentration measurements.

Year	Count	$\mathrm{Mean}\;\left(\mu g/m^{3}\right)$	$\mathrm{Median}\;\left(\mu g/m^{3}\right)$	$\mathrm{Standard}\;\mathrm{Deviation}\;\left(\mu g/m^{3}\right)$	Skewness	Kurtosis	$\mathrm{Minimum}\;\left(\mu g/m^{3}\right)$	$\mathrm{Maximum}\;\left(\mu g/m^{3}\right)$	Outliers %
$2008\;\left(X_{1}\right)$	8420	30.6714	29.190	13.8055	0.7028	3.7409	1.57	104.06	1.90
$2009\;\left(X_{2}\right)$	8463	29.0274	27.510	13.5032	0.8793	4.7740	1.39	121.16	1.86
$2010\;\left(X_{3}\right)$	8568	29.1409	27.970	13.0294	0.7802	4.5930	1.71	122.61	1.75
$2011\;\left(X_{4}\right)$	8462	27.4369	26.010	11.8807	0.6098	3.5480	1.46	84.99	1.67
$2012\;\left(X_{5}\right)$	8591	24.6597	23.380	11.8465	0.6422	3.4568	0.42	81.60	1.56
$2013\;\left(X_{6}\right)$	8288	28.0209	26.835	12.4174	0.8509	5.0159	1.81	114.84	1.68
$2014\;\left(X_{7}\right)$	8647	27.9431	26.430	12.8477	0.8255	4.9352	2.08	149.67	1.54
$2015\;\left(X_{8}\right)$	8529	27.0562	25.130	13.4024	0.8700	4.1198	0.29	110.47	2.03
$2016\;\left(X_{9}\right)$	8496	26.8132	25.610	12.8345	0.6392	3.6433	0	100.45	1.33
$2017\;\left(X_{10}\right)$	8282	23.4152	22.220	10.8507	0.7390	3.8362	0	88.03	1.79
$2018\;\left(X_{11}\right)$	8333	28.8103	27.180	12.7965	0.6763	3.5120	0	85.58	1.55
$2019\;\left(X_{12}\right)$	8474	25.9156	24.465	11.7287	0.6842	3.6442	2.17	89.66	1.33
Total	101,553	27.4108	25.950	12.7553	0.7747	4.2050	0	149.67	1.66

**Table 2:** Confidence interval limits for the median, with α = 0.05.

Variable	$\mathrm{Lower}\;\mathrm{Limit}\;\left(\mu g/m^{3}\right)$	$\mathrm{Upper}\;\mathrm{Limit}\;\left(\mu g/m^{3}\right)$
$X_{1}\;\left(2008\right)$	28.84	29.59
$X_{2}\;\left(2009\right)$	27.21	27.86
$X_{3}\;\left(2010\right)$	27.68	28.31
$X_{4}\;\left(2011\right)$	25.75	26.32
$X_{5}\;\left(2012\right)$	23.12	23.69
$X_{6}\;\left(2013\right)$	26.55	27.16
$X_{7}\;\left(2014\right)$	26.18	26.83
$X_{8}\;\left(2015\right)$	24.82	25.48
$X_{9}\;\left(2016\right)$	25.30	25.98
$X_{10}\;\left(2017\right)$	21.91	22.47
$X_{11}\;\left(2018\right)$	26.79	27.53
$X_{12}\;\left(2019\right)$	24.19	24.75

**Table 3:** Point estimates of location $\left(\mu g/m^{3}\right)$.

Year	Mean	$\mathrm{Median}\;m_{e}$	Trimean Tm	$0.2-\mathrm{Trimmed}\;\mathrm{Mean}\;T\left(0.2\right)$	$0.3-\mathrm{Trimmed}\;\mathrm{Mean}\;T\left(0.3\right)$	$0.2-\mathrm{Winsorized}\;\mathrm{Mean}\;W\left(0.2\right)$	$0.3-\mathrm{Winsorized}\;\mathrm{Mean}\;W\left(0.3\right)$	$\mathrm{Andrew}’s\;\mathrm{Wave}\;T_{wa}\left(2.4\pi \right)$	$\mathrm{Biweight}\;T_{bi}\left(9\right)$
$2008\;\left(X_{1}\right)$	30.6714	29.1900	29.4600	30.1907	28.3553	29.7156	29.4234	29.9821	29.9665
$2009\;\left(X_{2}\right)$	29.0274	27.5100	27.8175	27.4588	25.5255	28.0363	27.7640	28.2836	28.2726
$2010\;\left(X_{3}\right)$	29.1409	27.9700	28.1075	29.8856	29.1451	28.3203	28.0605	28.5222	28.5242
$2011\;\left(X_{4}\right)$	27.4369	26.0100	26.4650	26.9828	24.9782	26.7571	26.4540	26.9136	26.8660
$2012\;\left(X_{5}\right)$	24.6597	23.3800	23.6725	22.8323	20.3695	23.9037	23.6632	24.1463	24.1215
$2013\;\left(X_{6}\right)$	28.0209	26.8350	27.0750	27.9641	24.9496	27.2365	27.0573	27.4127	27.4106
$2014\;\left(X_{7}\right)$	28.0209	26.8350	27.0750	27.9641	24.9496	27.2365	27.0573	27.4127	27.4106
$2015\;\left(X_{8}\right)$	27.9431	26.4300	26.8250	25.8820	24.2028	27.1266	26.7925	27.3246	27.2912
$2016\;\left(X_{9}\right)$	27.0562	25.1300	25.6250	25.3397	22.3397	25.9492	25.5318	26.2370	26.1992
$2017\;\left(X_{10}\right)$	26.8132	25.6100	25.8300	24.5848	22.0544	26.0465	25.7487	26.3029	26.2872
$2018\;\left(X_{11}\right)$	23.4152	22.2200	22.4400	23.3262	23.3533	22.6466	22.3710	22.8758	22.8619
$2019\;\left(X_{12}\right)$	28.8103	27.1800	27.6150	28.3698	25.1177	27.9231	27.5771	28.2248	28.1775
All years	27.4108	25.9500	26.2850	27.0175	27.1500	26.5410	26.2272	26.7754	26.7526

**Table 4:** Point estimates of scale $\left(\mu g/m^{3}\right)$.

Year	$s_{x}$	$mAD_{mean}$	mAD	SRH	LmS	$s^{W}\left(0.2\right)$	$s_{wa}\left(2.4\pi \right)$	$s_{bi}\left(9\right)$	$C_{n}^{0.2}$
$2008\;\left(X_{1}\right)$	13.8055	10.8025	8.7000	8.7900	8.5300	8.3386	13.4431	13.4482	12.7180
$2009\;\left(X_{2}\right)$	13.5032	10.4953	8.5100	8.6250	8.4100	8.2029	12.8821	12.9066	12.5176
$2010\;\left(X_{3}\right)$	13.0294	10.1525	8.2800	8.3350	8.2150	7.9726	12.6009	12.6008	12.2625
$2011\;\left(X_{4}\right)$	11.8807	9.3367	7.4600	7.6000	7.3750	7.3040	11.7007	11.6932	11.0964
$2012\;\left(X_{5}\right)$	11.8465	9.3780	7.7500	7.8050	7.6050	7.4137	11.6913	11.6828	11.3332
$2013\;\left(X_{6}\right)$	12.4174	9.6233	7.9650	7.9850	7.8200	7.5782	11.8653	11.8836	11.7341
$2014\;\left(X_{7}\right)$	12.8477	10.0650	8.2400	8.4300	8.0400	7.9912	12.4651	12.4657	12.0803
$2015\;\left(X_{8}\right)$	13.4024	10.4634	8.4100	8.6400	8.0400	8.1693	12.8284	12.8536	11.9345
$2016\;\left(X_{9}\right)$	12.8345	10.1818	8.5650	8.6400	8.3300	8.2007	12.6827	12.6662	12.3354
$2017\;\left(X_{10}\right)$	10.8507	8.5253	6.9600	7.0100	6.7900	6.7041	10.5899	10.5901	10.1125
$2018\;\left(X_{11}\right)$	12.7965	10.1228	8.3600	8.5200	8.0150	8.0536	12.5949	12.5894	11.9163
$2019\;\left(X_{12}\right)$	11.7287	9.2944	7.6050	7.7100	7.5000	7.3684	11.5476	11.5364	11.3150
All years	12.7553	9.9982	8.1800	8.2700	8.0250	7.8793	12.3747	12.3789	11.9345

**Table 5:** 95% confidence intervals ($CI_{0.95}$ and confidence interval lengths: (*T*(0.2),*s^W^* (0.2)) and (*T_bi_* (9),*s_bi_* (9)).

Variable	$CI_{95}$	$\mathrm{Lower}\;\mathrm{Limit}\;\left(\mu g/m^{3}\right)$	$\mathrm{Upper}\;\mathrm{Limit}\;\left(\mu g/m^{3}\right)$	$\mathrm{Length}\;\left(\mu g/m^{3}\right)$
$X_{1}\;\left(2008\right)$	$\left(T\left(0.2\right),s^{W}\left(0.2\right)\right)$	29.8938	30.4876	0.5938
	$\left(T_{bi}\left(9\right),s_{bi}\left(9\right)\right)$	29.6949	30.2693	0.5745
$X_{2}\;\left(2009\right)$	$\left(T\left(0.2\right),s^{W}\left(0.2\right)\right)$	27.1675	27.7502	0.5827
	$\left(T_{bi}\left(9\right),s_{bi}\left(9\right)\right)$	28.0091	28.5581	0.5490
$X_{3}\;\left(2010\right)$	$\left(T\left(0.2\right),s^{W}\left(0.2\right)\right)$	29.6042	30.1670	0.5628
	$\left(T_{bi}\left(9\right),s_{bi}\left(9\right)\right)$	28.2554	28.7891	0.5337
$X_{4}\;\left(2011\right)$	$\left(T\left(0.2\right),s^{W}\left(0.2\right)\right)$	26.7233	27.2422	0.5189
	$\left(T_{bi}\left(9\right),s_{bi}\left(9\right)\right)$	26.6642	27.1629	0.4987
$X_{5}\;\left(2012\right)$	$\left(T\left(0.2\right),s^{W}\left(0.2\right)\right)$	22.5709	23.0936	0.5227
	$\left(T_{bi}\left(9\right),s_{bi}\left(9\right)\right)$	23.8990	24.3935	0.4945
$X_{6}\;\left(2013\right)$	$\left(T\left(0.2\right),s^{W}\left(0.2\right)\right)$	27.6921	28.2360	0.5440
	$\left(T_{bi}\left(9\right),s_{bi}\left(9\right)\right)$	27.1572	27.6682	0.5110
$X_{7}\;\left(2014\right)$	$\left(T\left(0.2\right),s^{W}\left(0.2\right)\right)$	25.6012	26.1627	0.5616
	$\left(T_{bi}\left(9\right),s_{bi}\left(9\right)\right)$	27.0618	27.5874	0.5256
$X_{8}\;\left(2015\right)$	$\left(T\left(0.2\right),s^{W}\left(0.2\right)\right)$	25.0507	25.6287	0.5780
	$\left(T_{bi}\left(9\right),s_{bi}\left(9\right)\right)$	25.9647	26.5093	0.5446
$X_{9}\;\left(2016\right)$	$\left(T\left(0.2\right),s^{W}\left(0.2\right)\right)$	24.2941	24.8755	0.5814
	$\left(T_{bi}\left(9\right),s_{bi}\left(9\right)\right)$	26.0332	26.5726	0.5395
$X_{10}\;\left(2017\right)$	$\left(T\left(0.2\right),s^{W}\left(0.2\right)\right)$	23.0855	23.5669	0.4814
	$\left(T_{bi}\left(9\right),s_{bi}\left(9\right)\right)$	22.6477	23.1040	0.4562
$X_{11}\;\left(2018\right)$	$\left(T\left(0.2\right),s^{W}\left(0.2\right)\right)$	28.0815	28.6580	0.5765
	$\left(T_{bi}\left(9\right),s_{bi}\left(9\right)\right)$	27.9544	28.4953	0.5410
$X_{12}\;\left(2019\right)$	$\left(T\left(0.2\right),s^{W}\left(0.2\right)\right)$	24.5174	25.0405	0.5231
	$\left(T_{bi}\left(9\right),s_{bi}\left(9\right)\right)$	25.1326	25.6244	0.4918
